# The Development of Gender Stereotypes About Service

**DOI:** 10.1111/desc.70298

**Published:** 2026-11-05

**Authors:** Sophie H. Arnold, Katharina Block, Andrei Cimpian

**Affiliations:** ^1^ Department of Psychology Princeton University Princeton New Jersey USA; ^2^ Department of Psychology University of Amsterdam Amsterdam Netherlands; ^3^ Department of Psychology New York University New York New York USA

**Keywords:** development, gender stereotypes, prosocial behavior, service

## Abstract

At work and at home, women perform more service than men, often at a personal cost. We investigated a psychological factor that may contribute to this disparity: an early‐emerging descriptive gender stereotype that associates service with girls. We compared 5‐ to 8‐year‐old children's (*N* = 192) and adults’ (*N* = 192) expectations about whether girls or boys would help across three types of scenarios: (a) interpersonal helping (which benefited a specific individual), (b) non‐costly service (which benefited a collective at no cost to the helper), and (c) costly service (which benefited a collective at a cost to the helper). Participants associated girls more than boys *with costly service specifically*. This association was especially noticeable among boys and men, who showed neutral expectations about interpersonal helping and non‐costly service but expected girls (vs. boys) to perform costly service. Girls and women showed a broader tendency to expect girls (vs. boys) to help across contexts. Separately, we also measured more general communality stereotypes (i.e., beliefs that girls are more other‐oriented than boys). These stereotypes predicted expectations about costly service but did not fully account for them: The association between girls and costly service persisted even after adjusting for individual differences in these stereotypes. These findings suggest that gendered expectations are strongest when helping demands personal sacrifice for a group or institution. These intensified expectations are already in place by ages 5 to 8, potentially representing one pathway through which gender disparities in service behavior develop over time.

## Summary

- Children 5 to 8 years of age and adults expect girls more than boys to perform costly service—helping the group at personal cost.
- These gendered expectations are specific to costly service; they do not extend to interpersonal helping or non‐costly service.
- Gendered expectations of costly service are related to, but not fully explained by, stereotypes associating girls with communality more broadly.
- These findings identify a potential developmental pathway whereby early stereotypes contribute to adult gender disparities in service.

## 1 The Development of Gender Stereotypes About Service

Human prosociality emerges early in development, with little evidence of sex differences in infancy through preschool (e.g., Margoni et al. 2023); yet, by adulthood, prosociality is gendered. A particularly stark gender difference is observed in *service*, a form of prosocial behavior performed by an individual to benefit a group or institution to which they belong, typically outside their formal role in that group or institution. For example, an employee might volunteer to take notes during a meeting, a task that benefits the team but is rarely formally assigned, or a family member might take charge of planning a holiday gathering that everyone benefits from but is not explicitly designated to anyone. In most settings, from family kitchens to schools and corporate boardrooms, women are more likely than men to take on service tasks, often at a personal cost (Babcock et al. 2017, 2022; Daminger 2019; Järvinen and Mik‐Meyer 2024; Kidder 2002; Misra et al. 2012; O'Meara et al. 2017; Thompson et al. 2020).

While the adult disparities in service are well‐documented, their origins remain unclear. Here, we examine a psychological factor that may contribute to this gender disparity: an early‐emerging descriptive gender stereotype^1^ that associates service with girls and women. The main goal of the present research is to investigate this hypothesized stereotype: Do people in fact associate service with girls more than boys, and if so, when does this association emerge in development? In what follows, we review three bodies of literature that bear on whether children and adults might hold gender stereotypes about service: the literatures on (a) gender stereotypes about communality, (b) expectations about interpersonal helping, and (c) the tendency to favor one's own gender group (gender ingroup bias). These literatures offer different, and in some cases competing, predictions that we test empirically.

### 1.1 Gender Stereotypes About Communality

One reason to expect service to be associated with girls and women is that they are stereotyped as more communal (that is, more oriented toward the needs and welfare of others) than boys and men (e.g., Block et al. 2025; Eagly et al. 2020; Koenig 2018; Liben and Bigler 2002; C. F. Miller et al. 2009). For example, Block et al. (2025) assessed children's gender stereotypes about communality using items such as, “Who do you think cares more about always being friendly to other people? Boys or girls?” and found that children older than about 6 years of age reliably associated communal values more with girls than with boys.

Service involves directing effort toward the needs of others, so it falls under the scope of communal behavior. Like most communal behaviors, it is also typically performed on an informal basis rather than being formally designated. Thus, one possibility is that service will be associated with girls and women (vs. boys and men) to a similar degree and on a similar age trajectory as other communal behaviors. On the other hand, service is a distinctive type of communal behavior: It benefits entire collectives or institutions rather than specific individuals. This distinctive feature often (though not always) makes service particularly *costly*: In and of itself, helping a collective may be more onerous and time‐consuming than helping an individual. It may also bring a lower probability of reward or recognition. When helping a specific person, there is a clear recipient, and with that, the prospect of recognition and relationship maintenance; in contrast, service is often invisible. If present, the element of cost might *heighten* the association between girls/women and service because cost entails setting aside one's immediate interests to benefit the collective—a low‐agency element, insofar as agency involves prioritizing one's own interests and goals.^2^ Because agency is stereotypically masculine (e.g., Eagly et al. 2020), costly service may seem particularly characteristic of girls and women and particularly unexpected from boys and men.

Research on service among adults is consistent with the possibility of a stereotype associating it with girls and women. In a set of experimental studies, Babcock et al. (2017) found that in mixed‐gender groups, women volunteered more than men to perform a task that benefits the group but that most people would rather someone else do; however, in same‐sex groups, men and women volunteered at similar rates, suggesting that the gap depends partly on the social expectations activated in mixed‐gender settings. In addition, when a third party had to select someone to ask, they asked women more often than men. Thus, when gender is salient, people appear more likely to expect women to volunteer their time and effort for tasks that benefit the group at the expense of their own priorities. Qualitative and quantitative work in academia tells a similar story: Women academics view service as more obligatory, whereas men are more likely to view it as not worth their time (Järvinen and Mik‐Meyer 2024; O'Meara et al. 2017).

Taken together, these considerations suggest the broad “communality = girls and women” association might well extend to service. They also raise the possibility that this association varies in strength for service versus other forms of communal behavior, as well as for different forms of service. Broad stereotypes about a domain do not necessarily extend equally to all parts of that domain. For example, although math and reading are both academic subjects, they evoke markedly different gender stereotypes (e.g., C. F. Miller et al. 2009; D. I. Miller et al. 2024; Wong et al. 2023). With respect to service, the association with girls and women may be heightened when service entails a cost because cost adds a low‐agency (specifically, self‐subordinating) element to an already communal behavior.

To examine these possibilities, in the present research we compared a prototypical form of communal behavior (helping another person) with two forms of service: non‐costly service (which benefits a collective without requiring a personal sacrifice) and costly service (which benefits a collective at a personal cost to the individual). This design allows us to test whether girls and women are more strongly associated with service than with more “ordinary” forms of communal behavior, and whether this association is especially pronounced when service entails a cost.

### 1.2 Expectations About Interpersonal Helping

A different perspective on children's expectations about who performs service emerges from research on interpersonal helping. This literature suggests an organizing principle based not on the gender of the helper but on the relationship between helper and recipient. Children generally expect helping to occur more often between similar or otherwise socially connected individuals. For example, children expect an individual's friends and family members to help them more than they expect strangers to help (Marshall et al. 2022; Yang et al. 2023). They also expect individuals to help same‐race more than different‐race others (Renno and Shutts 2015), and even expect helping to preferentially occur between members of minimal groups (Dunham et al. 2011; Rhodes 2012). Consistent with this broader pattern, children also expect more helping between members of the same gender group (Renno and Shutts 2015; Weller and Lagattuta 2014). Renno and Shutts (2015) examined 3‐ to 5‐year‐olds’ expectations of who would help them, a girl or a boy, and found that children expected more same‐gender helping than different‐gender helping, with this expectation being stronger among girls than among boys. Similarly, Weller and Lagattuta (2014) found that 5‐ to 13‐year‐olds expected more same‐gender helping than other‐gender helping, with no significant differences between girls and boys.

Because service is directed toward a collective rather than an identifiable individual recipient, it is not clear that the same‐gender similarity principle observed in interpersonal helping extends straightforwardly to service. Research on interpersonal helping therefore yields no clear prediction about which gender children should expect to perform service. It does, however, suggest that we should *not* observe a generalized expectation that girls perform service if children's judgments are driven primarily by similarity between helpers and recipients.

### 1.3 Gender Ingroup Bias

Children exhibit strong own‐gender positivity biases that may shape their expectations about who provides service. Preschool‐ and elementary school‐aged children generally attribute positive traits to their gender ingroup (Martin and Ruble 2010; C. F. Miller et al. 2006), although this broad positivity becomes more nuanced with age and differs in magnitude across gender groups. In a large sample spanning ages 5–17 and adults, both girls and boys implicitly associated their own (vs. the other) gender with positive words (e.g., “good”) more than negative words (e.g., “bad”), but this bias was reliably stronger among girls than among boys; the bias further attenuated with age among males, such that adult men showed no implicit own‐gender positivity (Dunham et al. 2016; see also Rudman and Goodwin 2004). This weaker ingroup‐positivity bias among boys (vs. girls) may reflect positive societal stereotypes about girls and women, particularly with respect to communal characteristics such as warmth, kindness, and nurturance (e.g., the “women are wonderful” effect; Eagly and Mladinic 1994). The further attenuation of ingroup positivity with age among males may reflect an additional process: increasingly negative associations attached to boys and men over age, including stereotypes linking males with aggression and violence (Dunham et al. 2016; Rudman and Goodwin 2004).

If expectations about service reflect this broader ingroup‐positivity bias, children should favor their own gender in their expectations: Girls should expect girls to perform more service, and (perhaps to a lesser degree) boys should expect boys to perform more service. On this account, then, children's expectations should track the perceiver's gender rather than uniformly favor girls. If children of both genders instead show a consistent expectation that girls will perform more service than boys, this pattern would be difficult to explain in terms of ingroup positivity alone. Because own‐gender positivity appears weaker in adulthood, especially among men (e.g., Dunham et al. 2016), this account makes a weaker and less symmetric prediction for adults’ service expectations—the attenuation of ingroup bias may allow other gender‐linked beliefs, such as stereotypes associating service with women, to shape expectations more readily.

### 1.4 Prior Developmental Evidence

The three accounts reviewed above generate predictions about children's expectations of who performs service. Although no prior work has tested these expectations directly, one recent study explored two related constructs: children's own service behavior and their prescriptive stereotypes about whether girls or boys *should* perform service. In a sample of 6‐ to 9‐year‐olds, Arnold and Rhodes (2026) found that, by age 9, girls were more likely than boys to choose to perform service. However, this behavioral difference did not correspond to a difference in children's prescriptive stereotypes: Children thought that girls and boys should perform service equally. This dissociation raises an important possibility. If girls and boys differ in their willingness to perform service even when children endorse egalitarian beliefs about who *should* do so, prescriptive stereotypes about service in general cannot account for the emerging behavioral difference. Descriptive stereotypes (that is, expectations about who *actually* does perform service) may also play a role and are our focus here.

The present study extends this prior work in two additional ways. First, Arnold and Rhodes (2026) examined only costly service (that is, they did not manipulate the cost aspect), leaving open the question of whether personal cost is necessary for service to become gendered. Second, we examine children at ages preceding the point at which Arnold and Rhodes observed a gender difference in behavior. If descriptive gender stereotypes about service contribute to the emergence of gender differences in service behavior, then these stereotypes should be detectable before the corresponding differences in behavior emerge, consistent with knowledge–behavior gaps documented in other domains of prosocial behavior (e.g., Amir et al. 2026).

### 1.5 The Present Research

In the present study, we examined whether 5‐ to 8‐year‐old children and adults associate service with girls more than boys. Participants judged whether a girl or a boy would provide help across three conceptually distinct types of communal scenarios: (a) interpersonal helping (directed at an individual recipient), (b) non‐costly service (directed at a collective, without a personal cost), and (c) costly service (directed at a collective, requiring a personal sacrifice). We focused on the 5‐ to 8‐year‐old age range because it spans a developmental window during which ingroup bias is strong, and gender stereotypes about communality are emerging (e.g., Block et al. 2025), while also preceding the age at which prior work has detected a gender difference in children's own service behavior (Arnold and Rhodes 2026). This age range therefore allows us to ask whether descriptive stereotypes about service emerge before the corresponding behavioral differences are detectable. We examined adults’ expectations as well, not just children's, because adults’ gendered expectations of children may guide differential treatment of girls and boys, contributing to the socialization of gender differences in these behaviors.

The three bodies of literature reviewed above generate different, and in some cases competing, predictions about the pattern of responses we should observe. The communality literature predicts that children and adults will associate any type of helping with girls more than boys. Moreover, to the extent that costly service reflects a form of communality that is intensified by self‐sacrifice (that is, by subordinating one's own interests to others’), then the association with girls may be strongest for these scenarios. To further test whether any tendency to associate costly service with girls *exceeds* what would be expected from broader communality stereotypes alone, we also separately measured these stereotypes (Block et al. 2025) and tested whether the association between costly service and girls was still observed after accounting for them.

In contrast, the literature on children's expectations about interpersonal helping yields no clear prediction about service. Because these expectations are organized around similarity within identifiable dyads, they do not straightforwardly extend to contexts in which help benefits a collective rather than an individual. This literature does, however, suggest that we should not observe a general tendency to expect girls rather than boys to help across contexts.

A third account, ingroup positivity, predicts that children will favor their own gender across all three scenarios, all of which involve positive behaviors, with this bias being stronger among girls than among boys (e.g., Dunham et al. 2016). Among adults, particularly men, ingroup positivity is weaker (e.g., Dunham et al. 2016; Rudman and Goodwin 2004), potentially allowing stereotypes linking (costly) service with girls and women to emerge more clearly.

## 2 Method

### 2.1 Open and Transparent Scientific Practices

The research questions, methods, exclusion criteria, and analyses for the child data were preregistered on AsPredicted.org (https://aspredicted.org/ccm4‐nhph.pdf; amendment: https://aspredicted.org/hv56‐kvs9.pdf). We registered a two‐step sequential analysis, with stopping points at *N* = 128 and 192; this method allows interim analyses at a prespecified point when data collection is costly, adjusting the *α* level to keep overall Type I error in check (see Lakens 2014). The results of our analyses at *N* = 128 led us to continue collecting data until we reached *N* = 192, where we used an adjusted *α* level of 0.0303. After collecting the child data, we collected a matched sample of 192 adults. We did not preregister this sample separately, relying instead on the preregistration for the child sample. The raw data and analytic scripts for both samples are shared on the Open Science Framework (OSF): https://osf.io/7cuj2/overview?view_only=f1c8a17b564142e2b17400ed81ca6fdd. All deviations from the preregistration can be found in Table S1 in the SI.

### 2.2 Participants

The final child sample consisted of 192 children between the ages of 5 and 8 years (*M*_age_ = 7.0 years, *SD*_age_ = 1.2; 50% girls, 50% boys). An additional 37 children were excluded due to the following preregistered reasons: failing 50% or more comprehension check questions (*n* = 29), completing less than 50% of the survey (*n* = 7), and parental interference (*n* = 1). We also tested one gender‐nonbinary participant but were unable to include them in our analyses since, as preregistered, our main models included a term for participant gender, and we did not have enough nonbinary children to form a separate gender category. Among the 86% of children whose parents reported their racial and ethnic identity, 68% were White, 13% Asian, 2% Black, and 16% multiracial; 7% were Hispanic and 93% were non‐Hispanic.

The final adult sample consisted of 192 adults; 98 were recruited through a university subject pool (*n* = 98; *M*_age_ = 19.9 years, *SD*_age_ = 1.3; 53.1% women, 46.9% men) and 94 were recruited through Prolific (*n* = 94; *M*_age_ = 34.8 years, *SD*_age_ = 11.0; 58.5% women, 41.5% men). An additional 19 participants were excluded for the following reasons: failing 50% or more of the comprehension checks (*n* = 17) and completing less than 50% of the study (*n* = 2). An additional five gender‐nonbinary individuals also participated, but we could not use their data for the reasons above. All but one participant reported their race; 49.7% were White, 38.2% Asian, 7.3% Black, 4.2% bi‐ or multiracial, and <1% American Indian or Alaskan Native. All participants reported their ethnicity; 13.0% were Hispanic or Latino, and 87.0% were not Hispanic or Latino.

### 2.3 Power Analysis

We conducted a simulation‐based sensitivity power analysis using the *mixedpower* package version 0.1.0 (Kumle et al. 2021) in R version 4.3.1 (R Core Team 2023), with *α* = 0.05 (two‐sided) and 1000 iterations, based on the planned mixed‐effects regressions and the observed random‐effects variances (see Results section for details). Our sample provided approximately 80% power to detect differences between conditions (e.g., costly service vs. interpersonal helping) as small as standardized *β* = 0.20–0.21, which is conventionally a small effect (Cohen 1992).

### 2.4 Procedure and Materials

The procedure for child and adult participants was largely the same, with minor differences to allow adult participants to complete the study independently. Children had the script read aloud by an experimenter, whereas adults read the study materials and answered the questions on their own.

The design was fully within‐subject. Participants read six vignettes about children in school (two each about interpersonal helping, non‐costly service, and costly service) and indicated whether they thought a girl or a boy would help in each scenario. There were no warm‐up trials. The order of the costly and non‐costly service vignette pairs was counterbalanced, while the two interpersonal helping vignettes were always presented last to avoid anchoring participants’ expectations about service on a more familiar form of helping (see Figure 1). After responding to the six vignettes, participants reported their gender stereotypes about communality. Finally, they completed a behavioral measure of costly helping (see Section 7 in the SI for details on this measure and results).

**FIGURE 1:**
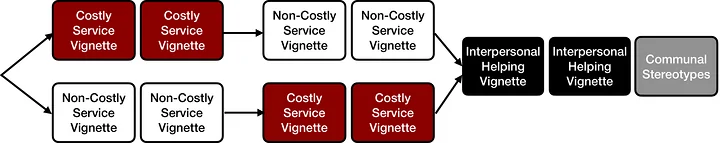
Overview of study procedure*. Note*: Participants completed four service vignettes, with the order of costly and non‐costly service blocks counterbalanced across participants, followed by two interpersonal helping vignettes and the measure of communal stereotypes. Participants also completed a behavioral measure of costly interpersonal helping at the end of the study (not shown, but see Section 7 of the SI).

#### 2.4.1 Service Vignettes

Four vignettes described typical classroom situations that involve service: showing a new student around, setting up tables for a bake sale, setting up materials for a class project, and putting up posters for a book fair (see Section 1 in the SI for all vignettes). Each service vignette appeared in either a non‐costly or a costly version (never both for the same participant), such that participants judged two service behaviors in a non‐costly form and two in a costly form. Assignment of vignettes to the costly versus non‐costly version was counterbalanced across participants. Each vignette was accompanied by a picture of four racially diverse cartoon girls and four racially diverse cartoon boys, with the placement of the girl and boy groups (left vs. right) randomized across participants but consistent within participant. In all vignettes, the participant had to guess whether “one of the girls or one of the boys” performed the relevant service; the order in which the two groups were mentioned matched the left/right position of the groups.

The example below illustrates the key elements of the service vignettes. Brackets indicate the text that was edited to toggle between the costly and non‐costly versions of the sample vignette, with the underlined text used in the costly version and the plain text in the non‐costly version. First, participants read information about the general context in which the need for service arose:

All of these children are in class. [*costly*: They are all looking forward to playing with all of their friends at recess. / *non‐costly*: None of them are looking forward to recess because all their friends are home sick, so they'll have no one to play with.]

Participants then answered an attention check intended to verify whether they encoded the crucial information in brackets: “Can you remind me, were these children all looking forward to playing with their friends for recess? Or were they all not looking forward to recess because all their friends were home sick?” Participants had one chance to answer this question correctly and then, regardless of their answer, were told the correct information. If participants answered this question incorrectly, we dropped their response to that particular vignette from the dataset. If a participant answered two or more of these check questions incorrectly, we dropped all data from that participant (*n* = 29).

Next, participants were told about the service that was required: “Okay, now here's something else: There's a project later that day, and the teacher said it would be great for someone to help set up the materials instead of playing outside for recess.” Lastly, participants reported who they thought would perform the service, one of the girls or one of the boys, and their confidence in their choice:

One of these children said they would help set up the materials [*costly*: even though they wanted to play outside with their friends for recess / *non‐costly*: since they didn't want to play outside for recess anyway]. Which of these children said they would help set up the materials? One of the boys or one of the girls? [*After the initial answer*:] How sure are you that one of the [boys/girls] said they would help set up the materials? Are you sort of sure? Or really sure?

Responses to these vignettes were thus recorded on a four‐point scale ranging from −1.5 = *really sure one of the boys* to 1.5 = *really sure one of the girls*. Zero was the theoretical neutral midpoint of this scale, corresponding to no directional expectation (i.e., equal likelihood that a boy or a girl would do the service).

As preregistered, we averaged responses across vignettes of the same type to assess participants’ gender stereotypes about service, resulting in two scores: one for costly service and one for non‐costly service.

#### 2.4.2 Interpersonal Helping Vignettes

After the four service vignettes, participants were presented with two vignettes featuring interpersonal helping (order randomized; see Section 1 in the SI for the full text). In each of these vignettes, participants indicated who they thought would help a student in distress (specifically, a student who fell off a swing and a student who didn't have any food for lunch), one of the boys or one of the girls (−1.5 = *really sure one of the boys* to 1.5 = *really sure one of the girls*). The gender of the student in need was not specified. This was done to avoid effects of the recipient's gender (e.g., gender‐matching expectations such as boys helping boys), so that responses would reflect expectations about whether girls or boys are more likely to help in this interpersonal context regardless of the recipient's gender. We did not manipulate cost for these vignettes; thus, no attention check was administered. As with the service vignettes, we averaged participants’ responses across these two vignettes to create a single stereotype score for interpersonal helping.

#### 2.4.3 Gender Stereotypes About Communality

To measure participants’ gender stereotypes about communality, we adapted six items from Block et al. (2025). Participants reported whether they thought boys or girls cared more about (a) doing things together with other people, (b) always being kind to other people, (c) thinking about other people's feelings, (d) always being nice to other people, (e) always trying to make other people happy, and (f) always being friendly to other people. Participants responded on a five‐point scale ranging from −2 = *boys care a lot more* to 2 = *girls care a lot more*, with a neutral midpoint of 0 = *boys and girls care the same amount*. We averaged responses to the six items to create a composite index of gender stereotypes about communality (child participants: *α* = 0.77; adult participants: *α* = 0.71). Section 6 in the SI contains additional analyses of these measures, beyond those reported below. These analyses replicate the gender and age differences reported in prior work on children's stereotypes about communality (Block et al. 2025).

#### 2.4.4 Behavioral Measure of Costly Interpersonal Helping

Lastly, children and adults were given an opportunity to provide help to the researcher at a cost to their own time. We initially conceived of this measure as a behavioral analogue of costly service. It does not, however, meet the definition of service used in the present research: Participants were asked to help the experimenter—an individual—rather than to take on work that benefits a group or institution to which they belong. Thus, this measure is more appropriately described as costly interpersonal helping than as costly service. In addition to this conceptual issue, the helping behavior elicited was different in the child and adult samples, limiting its comparability across age groups. For these reasons, we do not include this measure in our primary analyses and instead report its procedures and analyses in full in Section 7 of the SI. This represents a deviation from the preregistered plan (see Table S1).

## 3 Results

### 3.1 Analytic Strategy

Our key question is whether 5‐ to 8‐year‐olds and adults associate service with girls more than boys. To answer this question, we conducted a linear mixed‐effects regression with vignette type (interpersonal helping [reference category], non‐costly service, costly service), participant age group (0 = *adults*, 1 = *children*),^3^ participant gender (0 = *boys/men*, 1 = *girls/women*), and all interactions predicting participants’ expectations about whether a boy or a girl would help (−1.5 = *really sure one of the boys* to 1.5 = *really sure one of the girls*). The model included a random intercept for participant to account for the within‐subject structure of the data (each participant responded to all three vignette types). We report model‐predicted means (also known as “marginal means”) and their 95% CIs; CIs that exclude 0 indicate that participants expected one gender to help more than the other. Whether such expectations reflect gender stereotypes, ingroup positivity, or both is addressed by examining how these expectations vary across vignette types, participant gender, and age group. The preregistered sequential sampling and analysis plan for the child data required that we use an adjusted *α* level of 0.0303. We apply this same conservative threshold (0.0303) to the combined analyses reported here. We calculated effect sizes by computing a separate model with a standardized version of the outcome, which allows coefficients (*β*) to be interpreted as standardized mean differences comparable to Cohen's *d*.

The terms of primary interest in this model are (a) the main effects and pairwise contrasts among vignette types, which indicate which types of helping behavior (if any) are especially associated with girls (vs. boys); (b) the vignette type × age group interactions, which test whether these patterns differ between children and adults; and (c) the vignette type × participant gender interactions, as well as the three‐way vignette type × age group × participant gender interactions, which test whether these patterns differ for male versus female participants and whether such moderation by participant gender is different for children versus adults. The full model output can be found in Section 3 of the SI.

### 3.2 Participants’ Gender Stereotypes About Service

We found a significant main effect of participant gender, *χ* ^2^(1) = 67.90, *p* < 0.001, *β* = 0.52. Overall, girls and women thought that one of the girls helped (*M* = 0.36, 95% CI [0.30, 0.41]), whereas boys and men showed gender‐neutral expectations (*M* = 0.02, 95% CI [−0.04, 0.07]). This gender difference was larger for child participants (*M*_girls_ = 0.38 vs. *M*_boys_ = −0.06; *β* = 0.66, *SE* = 0.09, *p* < 0.001) than adult participants (*M*_women_ = 0.33 vs. *M*_men_ = 0.09; *β* = 0.37, *SE* = 0.09, *p* < 0.001), as revealed by the two‐way interaction between participant gender and age group, *χ* ^2^(1) = 5.17, *p* = 0.023. This interaction likely reflects children's more pronounced own‐gender bias relative to adults (e.g., Dunham et al. 2016): Girls favored girls and boys slightly favored boys, with this divergence narrowing among adults.

Most relevant to our research question, we also observed a significant two‐way interaction between vignette type and participant gender, *χ* ^2^(2) = 15.63, *p* < 0.001 (see Figure 2).^4^ To unpack this interaction, we examined the pattern of expectations separately for male and female participants. Girls and women expected girls to help across all three scenarios, with predicted means above the neutral midpoint (0) for interpersonal helping, non‐costly service, and costly service alike (see Figure 2); the differences among the three vignette types were not significant (all pairwise *p*s > 0.035; adjusted *α* level = 0.0303). In other words, girls and women showed a broad expectation that girls would help more than boys, regardless of the type of helping—a pattern consistent with both ingroup positivity and endorsement of a broad “communality = girls” stereotype that does not single out service as distinctly feminine.

**FIGURE 2:**
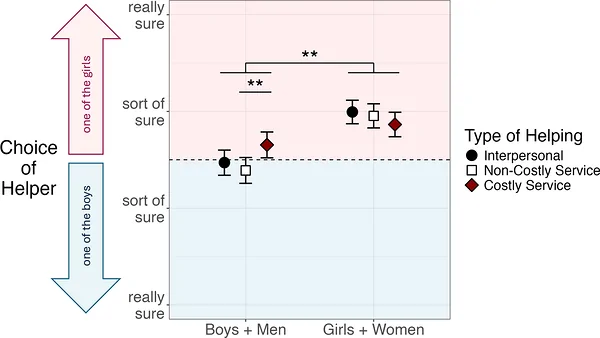
Participants’ Expectations about Helping Across Three Types of Scenarios: Interpersonal Helping, Non‐Costly Service, and Costly Service. *Note*: Shapes Are Predicted Means; Error Bars Are 95% Confidence Intervals. *p < 0.0303; **p < 0.01.

Boys and men showed a markedly different pattern. For interpersonal helping and non‐costly service, boys’ and men's expectations did not differ from the neutral midpoint (0), indicating no gender stereotype in either direction (see Figure 2). However, for costly service, boys and men expected girls to help significantly more than boys (*M* = 0.13, 95% CI [0.04, 0.23]). The contrast between costly service and non‐costly service (*M* = −0.01, 95% CI [−0.11, 0.08]) was significant (*β* = 0.22, *SE* = 0.10, *p* = 0.021), as was the contrast between costly service and interpersonal helping (*M* = −0.07, 95% CI [−0.17, 0.02]; *β* = 0.31, *SE* = 0.10, *p* = 0.001). Non‐costly service and interpersonal helping did not differ from each other (*β* = 0.09, *SE* = 0.10, *p* = 0.346). Thus, among male participants, the association between helping and girls emerged *only* for service that involved a personal cost.

There was no evidence that the overall pattern of responses varied by age group: Neither the vignette type × age group interaction, *χ* ^2^(2) = 1.65, *p* = 0.44, nor the three‐way vignette type × participant gender × age group interaction, *χ* ^2^(2) = 0.10, *p* = 0.95, was significant.

Additional exploratory (non‐preregistered) analyses revealed that the key interaction between vignette type and participant gender (a) was not moderated by the order in which the costly and non‐costly service vignettes were presented and (b) was also observed when the data were analyzed at the level of vignette (rather than averaged across the two vignettes of the same type; see Section 8 in the SI). Thus, the present findings do not appear to depend on incidental features of the design or analytic approach.

#### 3.2.1 Interim Summary

This pattern of results speaks directly to the competing predictions outlined in the introduction. The broad expectation among female participants that girls would provide more help than boys is consistent with both ingroup positivity and general communality stereotypes, which predict that girls will be seen as more other‐oriented than boys across contexts; the present data cannot distinguish between these accounts for female participants. The pattern among male participants is more theoretically diagnostic. They expected girls to help more in the costly service vignettes but were neutral in the other vignettes. This pattern is not readily explained by ingroup positivity, nor by a general “communality = girls” stereotype, which would predict a girl advantage across all three conditions. These results are instead most consistent with the possibility that costly service is perceived as especially communal because the helper's interests and goals are subordinated to those of a collective.

### 3.3 The Relation Between Communality Stereotypes and Service Stereotypes

If costly service is an intensified form of communality, then participants who more strongly endorse gender stereotypes about communality should also be more likely to expect girls (vs. boys) to perform costly service. At the same time, if stereotypes about costly service do capture something *beyond* “garden variety” other‐orientation, then communality stereotypes should not fully account for these stereotypes. That is, the expectation that girls rather than boys typically perform costly service should persist even after adjusting for individual differences in communality stereotypes.

We measured participants’ communality stereotypes using the scale from Block et al. (2025).^5^ We then added these stereotype scores, and their interactions with all other predictors, to the mixed‐effects model reported above. We focus our interpretation on the costly service scenarios because this is where a significant gender stereotype emerged among male participants.

The results indicated that communality stereotypes were positively related to participants’ expectations that girls (vs. boys) would perform costly service, *β* = 0.22, *SE* = 0.06, *p* < 0.001 (for full results, see Table S3 in the SI). However, even after adjusting for their communality stereotypes, both male (*M* = 0.20, 95% CI [0.10, 0.30]) and female (*M* = 0.25, 95% CI [0.15, 0.34]) participants still expected girls rather than boys to perform costly service.

#### 3.3.1 Interim Summary

Our results suggest that service stereotypes are rooted in, but also extend beyond, broader communality stereotypes. Although participants who more strongly associated girls with communality were also more likely to expect girls to perform costly service, this expectation remained reliable even after communality stereotypes were taken into account. This pattern suggests that people do not simply expect girls to be more other‐oriented in general; they also expect them, specifically, to bear personal costs for the collective.

## 4 Discussion

Women are expected to—and do—perform more service than men (Babcock et al. 2017, 2022; Daminger 2019; Järvinen and Mik‐Meyer 2024; Kidder 2002; O'Meara et al. 2017; Thompson et al. 2020). We investigated a psychological factor that may contribute to this gender difference: an early‐emerging descriptive stereotype about who performs service that associates these behaviors with girls. As an initial exploration of this stereotype, we measured 5‐ to 8‐year‐olds’ and adults’ expectations about whether girls or boys would help in contexts that called for interpersonal helping, non‐costly service, and costly service. Both children and adults, regardless of gender, thought that girls were more likely than boys to provide service when doing so came at a personal cost to the helper. Children's expectations did not vary with age and showed the same pattern across helping types as adults’, suggesting that this stereotype is already in place by age 5. This early‐emerging stereotype may be the developmental precursor of later expectations that women (rather than men) perform costly service at home and work (Babcock et al. 2017, 2022; Daminger 2019)—expectations that may contribute to women spending more time than men on service work throughout their lives.

Beyond this specific shared expectation about costly forms of service, however, the pattern diverged by participant gender. Girls and women expected girls to help more than boys across all three conditions, a pattern that can be explained both by ingroup positivity and by the presence of general communality stereotypes that associate girls with prosociality across contexts. Boys and men, in contrast, showed no expectation that girls would help more in interpersonal or non‐costly service contexts; this expectation emerged only for costly service. This selective pattern cannot be explained either by ingroup positivity on its own (which would predict own‐gender favoritism) or by broader communality stereotypes on their own (which would predict girl‐favoring expectations across all forms of helping). Instead, it suggests that for boys and men, general gender stereotypes about communality apply with special force (that can overpower ingroup biases) when communality takes an intensified form—namely, when helping requires setting aside one's own interests in favor of the collective's. This interpretation was further supported by our finding that endorsement of communality stereotypes predicted gendered costly‐service expectations but did not fully account for them: Even after adjusting for these broader beliefs, the tendency to associate costly service with girls remained robust.

### 4.1 Broader Theoretical and Methodological Implications

Research on gender and communality has often treated both communal behavior and the stereotypes surrounding it as monolithic categories: Girls and women are seen as more other‐oriented in a general sense (e.g., Block et al. 2025; Eagly et al. 2020; Koenig 2018). Consistent with this broader literature, and replicating Block et al. (2025), we found that both the older children and the adults in our sample associated communality more with girls than with boys (see Section 6 of the SI). Yet this broad belief did not translate into a uniform expectation that girls would help more than boys. Instead, that expectation emerged *specifically* when helping required absorbing a personal cost (specifically, setting aside one's goals) for the benefit of a collective. When helping was directed at an individual in need, or when it involved collective service without a personal sacrifice, boys and men showed no expectation that girls would help more. This dissociation suggests that gender stereotypes about communality are not organized solely around a diffuse belief that girls are more caring. These stereotypes also appear to encode a more specific expectation that girls and women will bear costs to sustain the functioning of groups and institutions.

The role of self‐sacrifice in our data reveals a parallel between the stereotypes observed here and Helgeson and Fritz's (1998) influential arguments about “unmitigated” communion, a focus on the needs of others to the exclusion of the self. According to Helgeson and Fritz, unmitigated communion is conceptually distinct from “standard” communion, which reflects warmth and interpersonal caring without implying low agency. The two are positively correlated but diverge sharply in their consequences: Standard communion is associated with satisfying relationships and psychological well‐being, whereas unmitigated communion predicts self‐neglect, difficulty with self‐assertion, and psychological distress. Critically for our purposes, what separates unmitigated communion from standard communion is not greater warmth or caring (i.e., high communality) but the willingness to absorb costs by placing others’ needs above one's own and having difficulty setting limits on demands from others. Our findings suggest a parallel distinction at the level of stereotypes: Just as unmitigated communion is related to but not reducible to communion as a personality trait, stereotypes about costly service are related to but not fully explained by broader stereotypes about communality. In light of this analogy, it is also noteworthy that the specific form of prosociality that children and adults consistently (regardless of gender) expect of girls and women is also central to a personality orientation associated with poorer psychological well‐being.

The analogy to unmitigated communion calls for precision about the relationship between costly service and agency. In the literature on agency and communion, one aspect of agency involves prioritizing and advancing one's own interests and goals (Abele and Wojciszke 2007; Bakan 1966). In this respect, costly service necessarily involves an element of reduced self‐prioritization: The helper gives up an immediate personal interest for the benefit of others. This does not mean, however, that costly service lacks agency in another sense of the term—intentional, goal‐directed action (e.g., Bandura 2001). People may choose to sacrifice for a group because they value its goals or believe they can make a difference. A further distinction concerns autonomy: Even when a sacrifice is intentional and goal‐directed, it may be freely chosen or made under pressure or obligation, with different consequences for the helper's well‐being (e.g., Weinstein and Ryan 2010). Our vignettes left the helper's motives unspecified: They established that the helper gave up something they wanted, not why. Future work could vary the helper's motives or measure participants’ inferences about them. The association between girls and costly service might hold regardless of motive, or it might be strongest when the sacrifice reflects pressure or obligation, which makes self‐subordination most salient. The answer would clarify whether the stereotype concerns who bears costs for the group or, more narrowly, who bears them under pressure.

Although the empirical pattern observed here will need to be replicated before firm conclusions can be drawn, it has implications for how developmental researchers study gender stereotypes of prosociality. Much of the existing literature asks, in one form or another, whether children view girls as more prosocial, usually by focusing on interpersonal helping (e.g., Weller and Lagattuta 2014). Our data suggest this question is underspecified. The answer may depend on the type of prosociality under investigation. The results presented here point to a potentially more informative framing: asking which forms of helping become gender‐typed and under what conditions. Questions of this kind require distinguishing among subtypes of prosocial behavior rather than treating them as interchangeable.

Because the vignettes about costly and non‐costly service were tightly matched except for the cost incurred by the protagonist (e.g., there was a benefit to the group in both, the role was informal in both, there was no recognition for the service in either), we are in a strong position to pinpoint cost as the “ingredient” that shifts expectations of helping toward girls, especially among boys and men. In other words, the female‐favoring descriptive stereotype documented here is not simply about who will help a collective or take on an informal helping role but more specifically about who will bear the costs of maintaining collective functioning, which is arguably a more consequential and burdensome expectation. Indeed, the adult literature has documented that women are disproportionately expected to accept “non‐promotable” tasks (Babcock et al. 2017, 2022), and that women academics experience service as more obligatory than men do (Järvinen and Mik‐Meyer 2024; O'Meara et al. 2017). Our findings extend this picture developmentally: Already among 5‐ to 8‐year‐olds, expectations about who will bear these costs resemble those observed among adults, suggesting that the gendered expectations implicated in later service disparities may have developmental roots that precede entry into the workforce by many years.

One might argue that there are also many roles that serve a collective but that are performed predominantly by men, including military service and political office (e.g., Eagly 2009; Heck et al. 2021). However, these roles are formally defined and publicly recognized and rewarded, whereas the service in our vignettes was informal and unrewarded. Thus, the expectation we document is not that women serve in the abstract but that they take on the personally costly, largely invisible work of maintaining the functioning of a group or institution. At the same time, this contrast highlights features that our design did not test. Cost is the only aspect of service we manipulated; formality and recognition were held constant, so our data cannot speak to what role they may play or to whether cost is sufficient on its own to elicit the stereotype. Future work varying these features alone and in combination could determine which are necessary and which are sufficient to give rise to the expectation that girls and women will take on service tasks.

Two features of our materials may have muted gendered expectations in the conditions where boys and men responded in a gender‐neutral manner. First, the non‐costly service scenarios did not rule out a personal benefit to the helper: The protagonists were explicitly described as bored when the need for service arose, so performing the service also relieved boredom. Future research could test whether it is the prospect of personal benefit or the absence of personal cost that leads to more gender‐neutral expectations. Second, one of the interpersonal helping vignettes involved saving a stranger from harm (specifically, a child who fell off the swings), a form of “heroic” helping that is more strongly associated with men than with women (e.g., Eagly 2009; Gutierrez et al. 2020; Hammond and Cimpian 2021). This association may have pulled expectations toward boys. Future research could test this possibility by comparing expectations across different forms of interpersonal helping (e.g., heroic rescue vs. everyday caregiving).

More broadly, boys’ and men's apparently gender‐neutral responses may not indicate an absence of gender‐related processes but rather the simultaneous operation of processes pushing in opposite directions. Own‐gender positivity favors choosing one's own gender (e.g., Dunham et al. 2016; Martin and Ruble 2010), whereas communality stereotypes favor choosing girls (e.g., Block et al. 2025; Eagly et al. 2020). These tendencies align for girls and women but conflict for boys and men, such that apparent gender neutrality among male participants may mask the operation of both. This account may also help explain why participant‐gender differences were larger among children than adults. Because own‐gender positivity weakens across development, particularly among males (Dunham et al. 2016), the tension between ingroup positivity and communality stereotypes may likewise weaken with age, producing less divergence between female and male participants. One further implication of this account is that ingroup positivity need not operate equally across all forms of helping: Its influence may depend on how positively the behavior itself is viewed. If boys and men regard costly service as less desirable than non‐costly service and interpersonal helping, they may be less inclined to associate it with their own gender, allowing the stereotype linking costly service with girls to emerge more clearly. Future work could test this possibility by examining how positively children and adults evaluate each type of helping.

A natural question prompted by these findings is how such specific expectations about costly service are learned in childhood. Perhaps the most plausible source is children's direct observation of gendered labor in their own homes (e.g., Daminger 2019). Midgette et al. (2023) found that children as young as 3 accurately reported that their mothers perform the majority of household labor. If children routinely see mothers, but not fathers, bearing the costs of keeping the family functioning, it would not be surprising for them to develop a generalized expectation that girls and women are the ones who typically bear such costs. More broadly, longitudinal work suggests that parents’ actual division of labor, more than their stated beliefs about gender, predicts children's later gender‐role attitudes (Croft et al. 2014; Cunningham 2001; Fulcher et al. 2008; Halpern and Perry‐Jenkins 2016), reinforcing the idea that what children see adults do shapes the expectations they form. The implication is that service stereotypes may not need to be explicitly taught; they may be learned through sustained observation of who, in children's daily environments, sacrifices personal time and energy for the group. This descriptive stereotype may also have *prescriptive* implications about who *should* perform costly service. In Midgette et al. (2023) study of perceptions of household labor, both children and their parents judged the unbalanced distribution they observed to be fair. This normalization of inequality is consistent with the broader “is‐to‐ought” tendency documented among both adults and children (e.g., Roberts et al. 2017; Tworek and Cimpian 2016). Extrapolating from this prior work, it is possible that children seamlessly transition from simply observing that women tend to do costly service work to inferring that this is how things should be.

### 4.2 Limitations and Future Directions

The present study represents a first step toward understanding the development of gender stereotypes about service. As an initial investigation, however, it has several limitations that future research should address.

Our primary focus was on descriptive gender stereotypes about service—that is, whether children and adults expect girls to perform more service than boys. Although we proposed that such stereotypes may represent one pathway through which gender differences in service emerge, we did not examine the relation between these stereotypes and participants’ own costly service behavior. Future research could directly test this link by providing children with opportunities to engage in service. Notably, recent work finds that gender differences in children's choice to perform service emerge only after age 9 (Arnold and Rhodes 2026). This pattern suggests that descriptive stereotypes may precede behavioral differences, consistent with evidence that expectations often guide subsequent behavior (e.g., Amir et al. 2026). Importantly, Arnold and Rhodes (2026) also found that older girls’ greater likelihood of choosing service was not explained by prescriptive beliefs—that is, girls did not report that girls should perform more service than boys. Together, these findings raise an interesting question: Is the descriptive‐to‐prescriptive transition necessary for stereotypes to shape behavior, or can descriptive beliefs alone channel children toward gendered patterns of service? Answering this question would clarify not only how service stereotypes operate but also where interventions might be most effectively targeted: at what children believe *is* the case versus *should be* the case.

Our study leaves open the question of whether gendered expectations are triggered by costliness alone or by costliness in combination with a collective target. Because we did not manipulate cost orthogonally with target (individual vs. collective)—that is, we did not include a costly interpersonal helping scenario—we cannot determine whether the “girls help more” expectation among boys and men would also emerge for costly interpersonal helping, or whether it is specific to costly service directed at a collective. A future design that crosses cost (low vs. high) with target (individual vs. collective) would allow researchers to isolate the independent contributions of each feature, clarifying whether girls are expected to deprioritize their own interests regardless of who benefits or only when doing so benefits a collective.

A separate but related question concerns how people determine whether a service behavior is costly in the first place. Our vignettes explicitly specified whether performing each service behavior involved a personal cost. Although vignette studies are useful for approximating the contexts in which children and adults make judgments in their everyday lives, people do not always have access to this information in real‐world contexts. Indeed, it can be difficult to know whether, or to what extent, performing an everyday service behavior carries a personal cost for another person. Thus, an important direction for future research is to examine how children and adults *infer* cost when it is not explicitly provided. For example, do they spontaneously assume that a boy (but not a girl) who performs a service behavior did so at little or no personal cost?

A further methodological consideration concerns the presentation order of the vignettes. The interpersonal helping vignettes were always presented after the four service vignettes. Thus, responses to the interpersonal helping scenarios may have been influenced by prior exposure to the service scenarios, and we cannot rule out this possibility with the present design. At the same time, the order in which participants encountered costly versus non‐costly service did not moderate the key interaction between vignette type and participant gender, providing some evidence that the central pattern of results was not driven by presentation order. Future work could fully counterbalance the placement of the interpersonal helping vignettes to test this possibility directly.

As already mentioned, the developmental origins of gender stereotypes about service remain an open question. One possibility is that they emerge through children's direct experiences with service in their everyday environments—for example, being asked to perform service or observing who performs service in their homes and classrooms (i.e., modeling; Bussey and Bandura 1999). If adults more frequently ask girls than boys to perform costly service tasks, children may detect this pattern and form corresponding descriptive stereotypes. A complementary possibility is that children observe broader gender differences in adult labor. Adult women perform more service than adult men (e.g., Daminger 2019), and children notice and accept this gendered distribution as fair from a young age (e.g., Midgette et al. 2023). This suggests that the home may be a context in which expectations about who bears collective costs are first learned. To date, however, the evidence linking parents’ division of labor to children's service expectations is limited and mixed. For instance, Cunningham (2001) found that parents’ division of housework when children were very young predicted children's own housework participation decades later, suggesting that early exposure to gendered labor can have lasting behavioral effects. In contrast, Arnold and Rhodes (2026) found that the proportion of housework reportedly completed by mothers (in heterosexual cohabiting couples) was unrelated to children's prescriptive beliefs about service or their own choices to perform service. It remains possible, however, that observing gendered labor shapes children's *descriptive* expectations (i.e., who usually does it), which were the focus of the present study, even if it does not immediately translate into prescriptive beliefs (i.e., who should do it) or personal preferences. An additional possibility is that, because adults—especially women, who hold this stereotype and are more represented in positions of power over children (e.g., as teachers)—believe girls do more service, they are more likely to ask girls than boys to perform this kind of costly work, which in turn makes girls more likely to engage in these behaviors later in childhood. Longitudinal work and experiments that manipulate children's exposure to who performs service (e.g., counter‐stereotypical models in home/classroom vignettes) would help clarify these pathways.

In adulthood, service burdens are stratified not only by gender but also by race and other intersecting identities. In the U.S., for example, both race and gender shape the distribution of service, with Black men and women performing more service than their White colleagues (Griffin and Reddick 2011; C. Miller and Roksa 2020). Thus, the stereotype we document here is likely embedded within a broader system of intersecting expectations about who will absorb collective costs. Although we used racially diverse cartoon images in our stimuli, our design did not allow us to examine whether gendered expectations about costly service vary as a function of race/ethnicity: that is, whether gendered expectations intersect with race/ethnicity such that expectations that girls perform more costly service than boys are more pronounced when thinking about some racial/ethnic groups compared to others. Relatedly, communal prescriptions for men are weaker in individualistic than collectivistic cultures (Kosakowska‐Berezecka et al. 2025), suggesting that the gender gap in communal expectations, and potentially in the costly service expectations documented here, may be wider in individualistic cultures such as the United States. Mapping how service stereotypes interact with race/ethnicity and cultural context will be essential for understanding whether these gendered expectations reflect a narrow or general phenomenon. More broadly, understanding the developmental emergence of intersectional stereotypes about service will be essential for understanding how early‐formed expectations contribute to the patterns of inequality observed in adulthood.

## 5 Conclusion

The present findings suggest that gender stereotypes about service are already in place by ages 5 to 8: Children and adults expected girls more than boys to bear personal costs for the benefit of a collective. Broader communality stereotypes were related to this expectation, but they did not fully account for it. These patterns indicate that communality stereotypes apply with special force when helping requires subordinating one's own goals to those of a group or institution. These early‐emerging stereotypes may be one source of the gendered distribution of service documented in families and workplaces.

## Author Contributions

**Sophie H. Arnold**: conceptualization, investigation, writing – original draft, methodology, visualization, writing – review and editing, formal analysis, project administration, data curation. **Katharina Block**: conceptualization, methodology, writing – review and editing. **Andrei Cimpian**: conceptualization, funding acquisition, writing – original draft, methodology, writing – review and editing, supervision, resources.

## Funding

This research was supported by New York University.

## Ethics Statement

This research received ethics approval from New York University's Institutional Review Board (IRB‐FY2016‐1163).

## Conflicts of Interest

The authors declare no conflicts of interest.

## Supporting information




**Supporting Information**: desc70298‐sup‐0001‐SuppMat.docx

## Data Availability

All data files and analytic scripts can be found on the Open Science Framework (OSF): https://osf.io/7cuj2/overview?view_only=f1c8a17b564142e2b17400ed81ca6fdd.

## Endnotes

1 By “descriptive gender stereotype,” we mean a belief about what girls/women and boys/men *typically* do, rather than a prescriptive belief about what they *should* do (e.g., Burgess and Borgida 1999). Although descriptive and prescriptive stereotypes are related, the present study focuses specifically on the former.

2 Definitions of agency vary: For example, some work distinguishes agency from competence (e.g., Eagly et al. 2020), whereas other work treats competence as a facet of agency (Abele et al. 2016). Our argument does not hinge on whether competence is considered part of agency; it concerns the self‐prioritization aspect of agency—advancing one's own interests and goals (Abele and Wojciszke 2007; Bakan 1966). Separately, although costly service requires setting aside an immediate personal interest to benefit others, it does not imply an absence of agency in the sense of intentional, goal‐directed action (e.g., Bandura 2001). We return to this latter distinction in the Discussion.

3 We first ran this model separately for child and adult participants (see Sections 4 and 5 of the SI, respectively). Because there were no age effects in the child sample, we report results for the combined dataset. The pattern of results was similar when the two samples were analyzed separately (see Figures S1 and S2 for visualizations of child and adult data modeled separately). The key interaction between participant gender and vignette type was significant for the adult sample (*p* = 0.008) but did not reach significance for the child sample (*p* = 0.033; adjusted *α* threshold = 0.0303).

4 As a robustness check, we tested whether performance on the comprehension checks (passing all four checks vs. only three) moderated the effects in the main model. There was no evidence that it did (*p*s > 0.07; see Section 8 in the SI), suggesting that participants who missed one comprehension check did not show systematically different response patterns from those who passed all four checks.

5 We replicated the findings from Block et al. (2025) pertaining to the age trajectory of communal stereotypes: Younger children in our sample held stronger gender ingroup biases, while older children and adults stereotyped girls as more communal than boys (see Section 6 in the SI for additional details and Figure S3 for visualization).

## References

[desc70298-bib-0001] Abele, A. E. , N. Hauke , K. Peters , E. Louvet , A. Szymkow , and Y. Duan . 2016. “Facets of the Fundamental Content Dimensions: Agency With Competence and Assertiveness—Communion With Warmth and Morality.” Frontiers in Psychology 7: 1810. 10.3389/fpsyg.2016.01810. PMC5118442 27920737

[desc70298-bib-0002] Abele, A. E. , and B. Wojciszke . 2007. “Agency and Communion From the Perspective of Self versus Others.” Journal of Personality and Social Psychology 93, no. 5: 751–763. 10.1037/0022-3514.93.5.751. 17983298

[desc70298-bib-0003] Amir, D. , R. E. Ahl , M. R. Jordan , et al. 2026. “The Emergence of Cooperative Behaviors, Norms, and Strategies Across Five Diverse Societies.” Science Advances 12, no. 6: eadw9995. 10.1126/sciadv.adw9995. PMC12880527 41650259

[desc70298-bib-0004] Arnold, S. H. , and M. Rhodes . 2026. “Who Should Do and Who Chooses to Do Service: Girls' and Boys' Developing Service‐Related Beliefs and Behaviors.” Child Development 97, no. 1: 97–110. 10.1093/chidev/aacaf008. 41543869

[desc70298-bib-0005] Babcock, L. , B. Peyser , L. Vesterlund , and L. Weingart . 2022. No Club: Putting a Stop to Women's Dead‐End Work. Simon and Schuster.

[desc70298-bib-0006] Babcock, L. , M. P. Recalde , L. Vesterlund , and L. Weingart . 2017. “Gender Differences in Accepting and Receiving Requests for Tasks With Low Promotability.” American Economic Review 107, no. 3: 714–747.

[desc70298-bib-0007] Bakan, D. 1966. Duality of human Existence: An Essay on Psychology and Religion. Rand McNally.

[desc70298-bib-0008] Bandura, A. 2001. “Social Cognitive Theory: An Agentic Perspective.” Annual Review of Psychology 52: 1–26. 10.1146/annurev.psych.52.1.1. 11148297

[desc70298-bib-0009] Block, K. , C. E. Hall , A. M. Gonzalez , A. Cimpian , T. Schmader , and A. S. Baron . 2025. “Who Cares About Caring? Gender Stereotypes About Communal Values Emerge Early and Predict Boys' prosocial Preferences.” Developmental Psychology 61, no. 3: 594–603. 10.1037/dev0001908. 39836163

[desc70298-bib-0010] Burgess, D. , and E. Borgida . 1999. “Who Women Are, Who Women Should be: Descriptive and Prescriptive Gender Stereotyping in Sex Discrimination.” Psychology, Public Policy, and Law 5, no. 3: 665–692. 10.1037/1076-8971.5.3.665.

[desc70298-bib-0011] Bussey, K. , and A. Bandura . 1999. “Social Cognitive Theory of Gender Development and Differentiation.” Psychological Review 106, no. 4: 676–713. 10.1037/0033-295x.106.4.676 10560326

[desc70298-bib-0012] Cohen, J. 1992. “A Power Primer.” Psychological Bulletin 112, no. 1: 155–159. 10.1037/0033-2909.112.1.155. 19565683

[desc70298-bib-0013] Croft, A. , T. Schmader , K. Block , and A. S. Baron . 2014. “The Second Shift Reflected in the Second Generation: Do Parents' Gender Roles at Home Predict Children's Aspirations?” Psychological Science 25, no. 7: 1418–1428. 10.1177/0956797614533968. 24890499

[desc70298-bib-0014] Cunningham, M. 2001. “The Influence of Parental Attitudes and Behaviors on Children's Attitudes Toward Gender and Household Labor in Early Adulthood.” Journal of Marriage and Family 63, no. 1: 111–122. 10.1111/j.1741-3737.2001.00111.x.

[desc70298-bib-0015] Daminger, A. 2019. “The Cognitive Dimension of Household Labor.” American Sociological Review 84, no. 4: 609–633. 10.1177/0003122419859007.

[desc70298-bib-0016] Dunham, Y. , A. S. Baron , and M. R. Banaji . 2016. “The Development of Implicit Gender Attitudes.” Developmental Science 19, no. 5: 781–789. 10.1111/desc.12321. 26260250

[desc70298-bib-0017] Dunham, Y. , A. S. Baron , and S. Carey . 2011. “Consequences of “Minimal” Group Affiliations in Children.” Child Development 82, no. 3: 793–811. 10.1111/j.1467-8624.2011.01577.x. PMC3513287 21413937

[desc70298-bib-0018] Eagly, A. H. 2009. “The His and Hers of Prosocial Behavior: An Examination of the Social Psychology of Gender.” American Psychologist 64, no. 8: 644–658. 10.1037/0003-066X.64.8.644. 19899859

[desc70298-bib-0019] Eagly, A. H. , and A. Mladinic . 1994. “Are People Prejudiced Against Women? Some Answers From Research on Attitudes, Gender Stereotypes, and Judgments of Competence.” European Review of Social Psychology 5, no. 1: 1–35. 10.1080/14792779543000002.

[desc70298-bib-0020] Eagly, A. H. , C. Nater , D. I. Miller , M. Kaufmann , and S. Sczesny . 2020. “Gender Stereotypes Have Changed: A Cross‐Temporal Meta‐Analysis of U.S. public Opinion Polls From 1946 to 2018.” American Psychologist 75, no. 3: 301–315. 10.1037/amp0000494. 31318237

[desc70298-bib-0021] Fulcher, M. , E. L. Sutfin , and C. J. Patterson . 2008. “Individual Differences in Gender Development: Associations With Parental Sexual Orientation, Attitudes, and Division of Labor.” Sex Roles 58, no. 5: 330–341. 10.1007/s11199-007-9348-4.

[desc70298-bib-0022] Griffin, K. A. , and R. J. Reddick . 2011. “Surveillance and Sacrifice: Gender Differences in the Mentoring Patterns of Black Professors at Predominantly White Research Universities.” American Educational Research Journal 48, no. 5: 1032–1057. 10.3102/0002831211405025.

[desc70298-bib-0023] Gutierrez, B. C. , M. L. D. Halim , M. A. Martinez , and M. Arredondo . 2020. “The Heroes and the Helpless: The Development of Benevolent Sexism in Children.” Sex Roles 82, no. 9–10: 558–569. 10.1007/s11199-019-01074-4.

[desc70298-bib-0024] Halpern, H. P. , and M. Perry‐Jenkins . 2016. “Parents' Gender Ideology and Gendered Behavior as Predictors of Children's Gender‐Role Attitudes: A Longitudinal Exploration.” Sex Roles 74, no. 11: 527–542. 10.1007/s11199-015-0539-0. PMC4945126 27445431

[desc70298-bib-0025] Hammond, M. D. , and A. Cimpian . 2021. “Wonderful but Weak”: “Children's Ambivalent Attitudes toward Women.” Sex Roles 84, no. 1–2: 76–90. 10.1007/s11199-020-01150-0.

[desc70298-bib-0026] Heck, I. A. , R. Santhanagopalan , A. Cimpian , and K. D. Kinzler . 2021. “Understanding the Developmental Roots of Gender Gaps in Politics.” Psychological Inquiry 32, no. 2: 53–71. 10.1080/1047840X.2021.1930741.

[desc70298-bib-0027] Helgeson, V. S. , and H. L. Fritz . 1998. “A Theory of Unmitigated Communion.” Personality and Social Psychology Review 2, no. 3: 173–183. 10.1207/s15327957pspr0203_2. 15647153

[desc70298-bib-0028] Järvinen, M. , and N. Mik‐Meyer . 2024. “Giving and Receiving: Gendered Service Work in Academia.” Current Sociology 00113921231224754. 10.1177/00113921231224754.

[desc70298-bib-0029] Kidder, D. L. 2002. “The Influence of Gender on the Performance of Organizational Citizenship Behaviors.” Journal of Management 28, no. 5: 629–648. 10.1177/014920630202800504.

[desc70298-bib-0030] Koenig, A. M. 2018. “Comparing Prescriptive and Descriptive Gender Stereotypes About Children, Adults, and the Elderly.” Frontiers in Psychology 9: 1086. https://www.frontiersin.org/articles/10.3389/fpsyg.2018.01086. 10.3389/fpsyg.2018.01086 PMC6028777 29997558

[desc70298-bib-0031] Kosakowska‐Berezecka, N. , A. Sawicki , G. Celikkol , et al. 2025. “Does Culture Moderate Gender Stereotypes? Individualism Predicts Communal (but Not Agentic) Prescriptions for Men Across 62 Nations.” Social Psychological and Personality Science 16, no. 4: 359–372. 10.1177/19485506231221913.

[desc70298-bib-0032] Kumle, L. , and M. L.‐H. Võ , and D. Draschkow . 2021. “Estimating Power in (generalized) Linear Mixed Models: An Open Introduction and Tutorial in R.” Behavior Research Methods 53, no. 6: 2528–2543. 10.3758/s13428-021-01546-0. PMC8613146 33954914

[desc70298-bib-0033] Lakens, D. 2014. “Performing High‐Powered Studies Efficiently With Sequential Analyses.” European Journal of Social Psychology 44, no. 7: 701–710. 10.1002/ejsp.2023.

[desc70298-bib-0034] Liben, L. S. , and R. S. Bigler . 2002. “The Developmental Course of Gender Differentiation: Conceptualizing, Measuring, and Evaluating Constructs and Pathways.” Monographs of the Society for Research in Child Development 67, no. 2: 1–183. 12465575

[desc70298-bib-0035] Margoni, F. , K. Block , K. Hamlin , N. Zmyj , and T. Schmader . 2023. “Meta‐Analytic Evidence Against Sex Differences in Infants' and Toddlers' Preference for Prosocial Agents.” Developmental Psychology 59, no. 2: 229–235. 10.1037/dev0001421. 36107660

[desc70298-bib-0036] Marshall, J. , A. Gollwitzer , K. Mermin‐Bunnell , M. Shinomiya , J. Retelsdorf , and P. Bloom . 2022. “How Development and Culture Shape Intuitions about Prosocial Obligations.” Journal of Experimental Psychology: General 151, no. 8: 1866–1882. 10.1037/xge0001136. 35130013

[desc70298-bib-0037] Martin, C. L. , and D. N. Ruble . 2010. “Patterns of Gender Development.” Annual Review of Psychology 61: 353–381. 10.1146/annurev.psych.093008.100511. PMC3747736 19575615

[desc70298-bib-0038] Midgette, A. J. , D. Ma , L. M. Stowe , and N. Chernyak . 2023. “US and Chinese Preschoolers Normalize Household Labor Inequality.” Proceedings of the National Academy of Sciences 120, no. 38: e2301781120. 10.1073/pnas.2301781120. PMC10515158 37695896

[desc70298-bib-0039] Miller, C. , and J. Roksa . 2020. “Balancing Research and Service in Academia: Gender, Race, and Laboratory Tasks.” Gender & Societ *y*, 34, no. 1: 131–152. 10.1177/0891243219867917.

[desc70298-bib-0040] Miller, C. F. , L. E. Lurye , K. M. Zosuls , and D. N. Ruble . 2009. “Accessibility of Gender Stereotype Domains: Developmental and Gender Differences in Children.” Sex Roles 60, no. 11: 870–881. 10.1007/s11199-009-9584-x. PMC2709873 19606278

[desc70298-bib-0041] Miller, C. F. , H. M. Trautner , and D. N. Ruble . 2006. “The Role of Gender Stereotypes in Children's Preferences and Behavior.” In Child Psychology: A Handbook of Contemporary Issues, 2nd ed., 293–323. Psychology Press.

[desc70298-bib-0042] Miller, D. I. , J. E. Lauer , C. Tanenbaum , and L. Burr . 2024. “The Development of Children's Gender Stereotypes about STEM and Verbal Abilities: A Preregistered Meta‐Analytic Review of 98 Studies.” Psychological Bulletin 150, no. 12: 1363–1396. 10.1037/bul0000456. 39652445

[desc70298-bib-0043] Misra, J. , J. H. Lundquist , and A. Templer . 2012. “Gender. Work Time, and Care Responsibilities among Faculty.” Sociological Forum 27, no. 2: 300–323. 10.1111/j.1573-7861.2012.01319.x.

[desc70298-bib-0044] O'Meara, K. , A. Kuvaeva , G. Nyunt , C. Waugaman , and R. Jackson . 2017. “Asked More Often: Gender Differences in Faculty Workload in Research Universities and the Work Interactions That Shape Them.” American Educational Research Journal 54, no. 6: 1154–1186. 10.3102/0002831217716767.

[desc70298-bib-0045] R. Core Team . 2023. *R: A Language and Environment for Statistical Computing* (Version 4.3.1) [Computer software]. R Foundation for Statistical Computing. https://www.R‐project.org/.

[desc70298-bib-0046] Renno, M. P. , and K. Shutts . 2015. “Children's Social Category‐Based Giving and Its Correlates: Expectations and Preferences.” Developmental Psychology 51, no. 4: 533–543. 10.1037/a0038819. 25706588

[desc70298-bib-0047] Rhodes, M. 2012. “Naïve Theories of Social Groups.” Child Development 83, no. 6: 1900–1916. 10.1111/j.1467-8624.2012.01835.x. 22906078

[desc70298-bib-0048] Roberts, S. O. , S. A. Gelman , and A. K. Ho . 2017. “So It Is, so It Shall be: Group Regularities License Children's Prescriptive Judgments.” Cognitive Science 41, no. Suppl 3: 576–600. 10.1111/cogs.12443. PMC5395313 27914116

[desc70298-bib-0049] Rudman, L. A. , and S. A. Goodwin . 2004. “Gender Differences in Automatic In‐Group Bias: Why Do Women like Women More than Men like Men?” Journal of Personality and Social Psychology 87, no. 4: 494–509. 10.1037/0022-3514.87.4.494. 15491274

[desc70298-bib-0050] Thompson, P. S. , D. M. Bergeron , and M. C. Bolino . 2020. “No Obligation? How Gender Influences the Relationship Between Perceived Organizational Support and Organizational Citizenship Behavior.” Journal of Applied Psychology 105, no. 11: 1338–1350. 10.1037/apl0000481. 32118460

[desc70298-bib-0051] Tworek, C. M. , and A. Cimpian . 2016. “Why Do People Tend to Infer “Ought.” From “Is”? The Role of Biases in Explanation.” Psychological Science 27, no. 8: 1109–1122. 10.1177/0956797616650875. 27485133

[desc70298-bib-0052] Weinstein, N. , and R. M. Ryan . 2010. “When Helping Helps: Autonomous Motivation for Prosocial Behavior and Its Influence on Well‐Being for the Helper and Recipient.” Journal of Personality and Social Psychology 98, no. 2: 222–244. 10.1037/a0016984. 20085397

[desc70298-bib-0053] Weller, D. , and K. H. Lagattuta . 2014. “Children's Judgments About Prosocial Decisions and Emotions: Gender of the Helper and Recipient Matters.” Child Development 85, no. 5: 2011–2028. 10.1111/cdev.12238. 24611809

[desc70298-bib-0054] Wong, W. I. , S. Y. Shi , and S. P. Yeung . 2023. “Girls Are Better Students but Boys Will be More Successful at Work: Discordance between Academic and Career Gender Stereotypes in Middle Childhood.” Archives of Sexual Behavior 52, no. 3: 1105–1121. 10.1007/s10508-022-02523-0. 36626072

[desc70298-bib-0055] Yang, F. , X. Yang , and Y. Dunham . 2023. “Beyond Our Tribe: Developing a Normative Sense of Group‐Transcendent Fairness.” Developmental Psychology 59, no. 7: 1203–1217. 10.1037/dev0001545. 37166870

