# Supplementary material for: The Development of Gender Stereotypes About Service
Source: Dev Sci. 2026 Nov 5;30(1):e70298. doi: 10.1111/desc.70298 (PMC13735205; doi:10.1111/desc.70298)
Supplement: Supplementary file 1 [file DESC-30-e70298-s001.docx]

Supplemental Materials for

The Development of Gender Stereotypes about Service

[Section 1: Vignettes	2](#_Toc237445888)

[Service Vignettes	2](#_Toc237445889)

[**Story 1: Show Student Around**	2](#_Toc237445890)

[**Story 2: Set Up Tables for Bake Sale**	3](#_Toc237445891)

[**Story 3: Set Up Materials for Project**	3](#_Toc237445892)

[**Story 4: Put up Posters for Book Fair**	4](#_Toc237445893)

[Interpersonal Vignettes	4](#_Toc237445894)

[**Story 1: Fell off Swings**	4](#_Toc237445895)

[**Story 2: No Lunch**	5](#_Toc237445896)

[Communal/Warmth Stereotype Items	5](#_Toc237445897)

[Behavioral Measure of Interpersonal Costly Helping	6](#_Toc237445898)

[**Child Item**	6](#_Toc237445899)

[**Adult Course Credit Item (University Subject Pool)**	7](#_Toc237445900)

[**Adult Monetary Compensation Item (Prolific)**	7](#_Toc237445901)

[Section 2: Deviations from Preregistration	8](#_Toc237445902)

[Section 3: Regression Tables for All Participants	10](#_Toc237445903)

[Section 4: Analyses with Only Child Participants	12](#_Toc237445904)

[Section 5: Analyses with Only Adult Participants	16](#_Toc237445905)

[Section 6: Communal Stereotypes	19](#_Toc237445906)

[Section 7: Costly Interpersonal Helping	20](#_Toc237445907)

[Section 8: Exploratory Robustness Checks	24](#_Toc237445908)

# Section 1: Vignettes

## Service Vignettes

[red text] = costly

[green text] = non-costly

Whether the group of boys or girls was presented on the left (vs. right) of the screen was randomized between participants and consistent within participants. The question text matched the order of the pictures. That is, when boys were presented on the left, they were referenced first “One of the boys or one of the girls?” while if girls were presented on the left, they were referenced first “One of the girls or one of the boys?”.

### **Story 1: Show Student Around**

     

All of these children are in class studying for their big test tomorrow. [They’re all worried about the big test tomorrow so they’re all studying really hard.] [They finished studying early so now they’re all just bored waiting for class to end.]

Comprehension Check: Can you remind me, were these children all worried about the test tomorrow? Or were they all bored waiting for class to end?

Okay, now here’s something else: There’s a new student in school and the teacher said it would be great for someone to help show the new student around.

One of these children said they would help show the new student around [even though they still need to study for the test tomorrow] [since they were done studying anyway]. Which of these children said they would help show the new student around? One of the boys or one of the girls?

How sure are you that one of the girls [boys] said they would help show the new student around? Are you sort of sure? Or really sure?

### **Story 2: Set Up Tables for Bake Sale**

     

All of these children are in class doing school work on their own. If they get their work done before the end of class, they don’t have to do any work at home! [They’re all working hard on their work so they can get it done before the end of class.] [They all just finished their work, so now they’re all just bored waiting for class to end.]

Comprehension Check: Can you remind me, were these children working hard on their work? Or were they all bored waiting for class to end?

Okay, now here’s something else: There’s a bake sale later that day and the teacher said it would be great for someone to help set up the tables for the bake sale.

One of these children said they would help set up the tables for the bake sale [even though they wanted to complete their work during class] [since they were all done with their work anyway]. Which of these children said they would help set up the tables? One of the boys or one of the girls?

How sure are you that one of the girls [boys] said they would help show the new student around? Are you sort of sure? Or really sure?

### **Story 3: Set Up Materials for Project**

     

All of these children are in class. [They are all looking forward to playing with all of their friends at recess.] [None of them are looking forward to recess because all their friends are home sick so they’ll have no one to play with.]

Comprehension Check: Can you remind me, were these children all looking forward to playing with their friends for recess? Or were they all not looking forward to recess because all their friends were home sick?

Okay, now here’s something else: There’s a project later that day and the teacher said it would be great for someone to help set up the materials instead of playing outside for recess.

One of these children said they would help set up the materials [even though they wanted to play outside with their friends for recess] [since they didn’t want to play outside for recess anyway]. Which of these children said they would help set up the materials? One of the boys or one of the girls?

How sure are you that one of the girls [boys] said they would help show the new student around? Are you sort of sure? Or really sure?

### **Story 4: Put up Posters for Book Fair**

     

All of these children are in class at the end of the school day. [All of these children’s parents are picking them up right after school. They are all excited to go home and spend time with their family.] [All of these children’s parents are running late to pick them up from school so they are all just bored waiting around.]

Comprehension Check: Can you remind me, were these children’s parents picking them up right after school? Or were all their parents running late?

Okay, now here’s something else: There’s a book fair later that night and the teacher said it would be great for someone to help put up posters for the book fair in the hallways.

One of these children said they would help put up posters [even though they wanted to go home right away] [since their parents were running late anyway]. Which of these children said they would help put up posters? One of the boys or one of the girls?

How sure are you that one of the girls [boys] said they would help show the new student around? Are you sort of sure? Or really sure?

## Interpersonal Vignettes

### **Story 1: Fell off Swings**

     

All of these children are at school and outside for recess. They’re all deciding what to do for recess.

All of these children saw that another student fell off the swings.

One of these children said they would help the fallen student off the playground and walk with them to the nurse’s office. Which of these children said they would help the fallen student? One of the boys or one of the girls?

How sure are you that one of the girls [boys] said they would help the fallen student? Are you sort of sure? Or really sure?

Comprehension Check: N/A

### **Story 2: No Lunch**

     

All of these children are at school eating lunch. They’re all eating the big lunches their parents packed for them.

All of these children saw that another student didn’t have any food to eat for lunch.

One of these children said they would help by giving half of their sandwich to the student who didn’t have any food. Which of these children said they would help the hungry student? One of the boys or one of the girls?

How sure are you that one of the girls [boys] said they would help the hungry student? Are you sort of sure? Or really sure?

Comprehension Check: N/A

## Communal/Warmth Stereotype Items

1. Who do you think cares more about **doing things together with other people**?
2. Who do you think cares more about **always being kind to other people**?
3. Who do you think cares more about **thinking about other people’s feelings**?
4. Who do you think cares more about **always being nice to other people**?
5. Who do you think cares more about **always trying to make other people happy**?
6. Who do you think cares more about **always being friendly to other people**?

Do boys care a lot more about [item]? Do boys care a little more? Do boys and girls care the same amount? Do girls care a little more? Or do girls care a lot more?

## Behavioral Measure of Interpersonal Costly Helping

### **Child Item**

Thank you so much for completing this activity! You know, a lot of other kids do this activity too. You know how you answered a lot of questions and chose either “boy” or “girl”? Well, they also answered all those questions! In order to understand what kids think, I need to count up everyone’s answers. That means, I need to count up all the times that kids chose “boy” and all the times that kids chose “girl.” It’s **a lot** of work!

What do you think, would you maybe be able to help me count up all the other kids’ answers right now or would you like me to ask someone else?

[If the child said they would help:] Great! How long do you think you could help me for? Only a short amount of time (like a couple of minutes), a medium amount of time (like thirty minutes) or a long amount of time (like an hour)?

Thank you so much! You know what? I actually almost forgot that I have another appointment right after yours! That means we actually can’t spend any time counting right now. Thank you so much for offering to help though, that is really nice of you!

### **Adult Course Credit Item (University Subject Pool)**

Thank you so much for completing this survey!

If you would like to, you can complete another survey to help us learn more about how people think about the world around them across development. If you don't want to complete this survey, you don't have to! Your 0.5 credit hours will be counted either way. This is an optional survey; you will not receive extra credit for completing this survey.

Would you be able to help us learn more and complete an additional survey or would you like to be done?

[If help:] Thank you! How long of an additional survey would you be interested in completing? Please remember that this is entirely voluntary, you'll get the same 0.5 credit hours regardless of what you choose. [3-5 minute survey, 10-15 minute survey, 25-30 minute survey]

### **Adult Monetary Compensation Item (Prolific)**

Thank you so much for completing this survey!

If you would like to, you can complete another survey to help us learn more about how people think about the world around them across development. If you don't want to complete this survey, you don't have to! Your payment will be counted either way. This is an optional survey; you will not receive extra payment for completing this survey.

Would you be able to help us learn more and complete an additional survey or would you like to be done?

[If help:] Thank you! How long of an additional survey would you be interested in completing? Please remember that this is entirely voluntary, you'll get the same payment regardless of what you choose. [3-5 minute survey, 10-15 minute survey, 25-30 minute survey]

# Section 2: Deviations from Preregistration

**Table S1**

*Deviations from Preregistration*

| **Preregistered Approach** | **Deviation in Current Manuscript** | **Rationale for Deviation** | **Impact on Interpretation** |
| --- | --- | --- | --- |
| **Planned analyses examining (a) service (costly + non-costly averaged) vs. interpersonal helping, and (b) costly vs. non-costly service separately** | Analyses instead model **three vignette types simultaneously** (interpersonal helping, non-costly service, costly service) in a single mixed-effects model | Averaging costly and non-costly service obscures the contrast that proved most informative: the selective emergence of stereotyping for costly service among male participants. A single model with three vignette types preserves all pairwise contrasts without the information loss inherent in collapsing across meaningfully different vignette types. | The joint model is more parsimonious: It includes fewer tests than the preregistered two-step approach. The key finding (stereotyping specific to costly service among male participants) would have been obscured under the preregistered averaging approach. Full preregistered analyses are available in the SOM and yield consistent conclusions. |
| **Structural equation model comparing one- vs. two-factor models to test whether service and communality stereotypes are distinct** | Instead of SEM model comparison, manuscript tests distinctiveness by adding communality stereotypes and their interactions into the mixed-effects model predicting helping expectations | The mixed-effects framework used for all other analyses does not easily accommodate latent variable modeling. A regression-based test directly addresses the theoretical question of whether communality stereotypes statistically account for service stereotypes, within the same analytic framework used throughout the manuscript. | This tests whether service stereotypes have incremental predictive validity beyond communality stereotypes, rather than testing whether they are structurally distinct latent factors. For the present purposes, this is the more relevant test: The theoretical question is not whether communality and service stereotypes load on different factors, but whether knowing someone’s communality stereotypes is sufficient to predict their service expectations. The answer is that it is not. |
| **Measure of behavioral costly helping included as a main dependent variable** | Results reported in SOM rather than the main text | On reflection, this measure does not adequately operationalize the construct of costly service as defined in the study (specifically, helping directed at a collective that entails a personal sacrifice). We report these analyses in the SOM (Section 7) for transparency and discuss the limitations of this measure there. | This deviation does not affect our conclusions about gender stereotypes about service, which are the primary focus of the manuscript. However, it limits our ability to understand how these stereotypes relate to children’s own service behaviors. |

*Note*. Preregistrations can be found on AsPredicted.org (<https://aspredicted.org/ccm4-nhph.pdf>; amendment: <https://aspredicted.org/hv56-kvs9.pdf>).

# Section 3: Regression Tables for All Participants

**Table S2**

*Linear Mixed-Effects Regression on Participants’ Expectations about Helping*

|  | **Expectations**  **about Helping** | | |
| --- | --- | --- | --- |
| *Predictors* | *χ*^2^ | *df* | *p* |
| Vignette Type | 0.51 | 2 | .776 |
| Female | 67.90 | 1 | **< .001** |
| Children | 1.03 | 1 | .310 |
| Vignette Type × Female | 15.63 | 2 | **< .001** |
| Vignette Type × Children | 1.65 | 2 | .439 |
| Female × Children | 5.17 | 1 | **.023** |
| Vignette Type × Female × Children | 0.10 | 2 | .951 |
| *N*_Participants_ | 384 | | |
| *N*_Observations_ | 1152 | | |

*Note*. Wald chi-square tests for the fixed effects of the linear mixed-effects model regressing children’s and adults’ expectations about helping on participants’ gender (Female; girls and women = 1, boys and men = 0), their age group (Children; children = 1, adults = 0), the type of helping (Vignette Type; interpersonal, non-costly service, costly service), and their interactions.

**Table S3**

*Linear Mixed-Effects Regression on Participants’ Expectations about Helping, Adjusting for Their Communal Stereotypes*

|  | **Expectations**  **about Helping** | | |
| --- | --- | --- | --- |
| *Predictors* | *χ*^2^ | *df* | *p* |
| Vignette Type | 0.51 | 2 | .776 |
| Female | 33.94 | 1 | **< .001** |
| Children | 1.14 | 1 | .286 |
| Communal | 51.58 | 1 | **< .001** |
| Vignette Type × Female | 12.19 | 2 | **.002** |
| Vignette Type × Children | 2.45 | 2 | .294 |
| Female × Children | 1.56 | 1 | .211 |
| Vignette Type × Communal | 4.55 | 2 | .103 |
| Female × Communal | 0.06 | 1 | .809 |
| Children × Communal | 3.43 | 1 | .064 |
| Vignette Type × Female × Children | 0.06 | 2 | .970 |
| Vignette Type × Female × Communal | 1.33 | 2 | .515 |
| Vignette Type × Children × Communal | 2.08 | 2 | .353 |
| Female × Children × Communal | 0.28 | 1 | .599 |
| Vignette Type × Female × Children × Communal | 0.79 | 2 | .675 |
| *N*_Participants_ | 384 | | |
| *N*_Observations_ | 1152 | | |

*Note*. Wald chi-square tests for the fixed effects of the linear mixed-effects model regressing children’s and adults’ expectations about helping on participants’ gender (Female; girls and women = 1, boys and men = 0), their age group (Children; children = 1, adults = 0), the type of helping (Vignette Type; interpersonal, non-costly service, costly service), their communal stereotypes (Communal; higher values correspond to stronger feminine stereotypes about communality), and all interactions.

# Section 4: Analyses with Only Child Participants

**Table S4**

*Means and Correlations Among All Measured Variables for Girls (Lower Left Triangle) and Boys (Upper Right Triangle)*

|  | *Costly Service* | *Non-costly Service* | *Interpersonal Helping* | *Communal Stereotypes* |
| --- | --- | --- | --- | --- |
| **Means** |  |  |  |  |
| *Girls* | 0.27 | 0.42 | 0.45 | 0.53 |
| *Boys* | 0.04 | −0.09 | −0.12 | 0.07 |
| **Correlations** |  |  |  |  |
| *Costly Service* | — | .10 | .14 | .27^**^ |
| *Non-costly Service* | .20 | — | .27^**^ | .18 |
| *Interpersonal Helping* | .25^*^ | .10 | — | .26^**^ |
| *Communal Stereotypes* | .16 | .24^*^ | .41^***^ | — |

*Note*. Pearson correlations on complete cases. The lower left triangle are correlations for girl participants; the upper right triangle are correlations for boy participants. Costly service, non-costly service, and interpersonal helping were measured on a 4-point scale ranging from −1.5 = *really sure one of the boys* to +1.5 *= really sure one of the girls.* Communal stereotypes were measured on a 5-point scale ranging from −2 = *boys care a lot more* to +2 = *girls care a lot more*. ^*^ *p* < .05, ^**^ *p* < .01, ^***^ *p* < .001

**Table S5**

*Linear Mixed-Effects Regression on Children’s Expectations about Helping*

|  | **Children’s Expectations  about Helping** | | |
| --- | --- | --- | --- |
| *Predictors* | *χ*^2^ | *df* | *p* |
| Vignette Type | 0.05 | 2 | .975 |
| Girl | 38.12 | 1 | **< .001** |
| Age | 0.06 | 1 | .805 |
| Vignette Type × Girl | 6.82 | 2 | .033 |
| Vignette Type × Age | 0.58 | 2 | .748 |
| Girl × Age | 3.46 | 1 | .063 |
| Vignette Type × Girl × Age | 1.73 | 2 | .421 |
| *N*_Participants_ | 192 | | |
| *N*_Observations_ | 576 | | |

*Note*. Wald chi-square tests for the fixed effects of the linear mixed-effects model regressing children’s expectations about helping on their gender (Girl; girls = 1, boys = 0), their age (Age; continuous, 5.01−8.98), the type of helping (Vignette Type; interpersonal, non-costly service, costly service), and their interactions.

**Figure S1**

*Children’s Expectations about Helping Across Three Types of Scenarios: Interpersonal Helping, Non-Costly Service, and Costly Service*

*Note*. Shapes are predicted means; error bars are 95% confidence intervals.

**Table S6**

*Linear Mixed-Effects Regression on Children’s Expectations about Helping, Adjusting for Their Communal Stereotypes*

|  | **Children’s Expectations**  **about Helping** | | |
| --- | --- | --- | --- |
| *Predictors* | *χ*^2^ | *df* | *p* |
| Vignette Type | 0.05 | 2 | .975 |
| Girl | 18.31 | 1 | **< .001** |
| Age | 0.03 | 1 | .874 |
| Communal | 28.77 | 1 | **< .001** |
| Vignette Type × Girl | 4.21 | 2 | .122 |
| Vignette Type × Age | 1.15 | 2 | .564 |
| Girl × Age | 0.38 | 1 | .538 |
| Vignette Type × Communal | 2.96 | 2 | .227 |
| Girl × Communal | 0.02 | 1 | .882 |
| Age × Communal | 1.02 | 1 | .312 |
| Vignette Type × Girl × Age | 1.16 | 2 | .559 |
| Vignette Type × Girl × Communal | 1.85 | 2 | .397 |
| Vignette Type × Age × Communal | 3.40 | 2 | .183 |
| Girl × Age × Communal | 0.91 | 1 | .341 |
| Vignette Type × Girl × Age × Communal | 0.86 | 2 | .651 |
| *N*_Participants_ | 192 | | |
| *N*_Observations_ | 576 | | |

*Note*. Wald chi-square tests for the fixed effects of the linear mixed-effects model regressing children’s expectations about helping on their gender (Girl; girls = 1, boys = 0), their age (Age; continuous, 5.01−8.98), the type of helping (Vignette Type; interpersonal, non-costly service, costly service), their communal stereotypes (Communal; higher values correspond to stronger feminine stereotypes about communality), and all interactions.

# Section 5: Analyses with Only Adult Participants

**Table S7**

*Means and Correlations Among All Measured Variables for Women (Lower Left Triangle) and Men (Upper Right Triangle)*

|  | *Costly Service* | *Non-costly Service* | *Interpersonal Helping* | *Communal Stereotypes* |
| --- | --- | --- | --- | --- |
| **Means** |  |  |  |  |
| *Women* | 0.29 | 0.34 | 0.36 | 0.81 |
| *Men* | 0.23 | 0.06 | −0.03 | 0.45 |
| **Correlations** |  |  |  |  |
| *Costly Service* | — | −0.17 | −0.04 | 0.28^**^ |
| *Non-costly Service* | 0.03 | — | −0.01 | 0.06 |
| *Interpersonal Helping* | 0.03 | 0.02 | — | 0.21 |
| *Communal Stereotypes* | 0.19 | 0.06 | 0.18 | — |

*Note*. Pearson correlations on complete cases. The lower left triangle are correlations for women; the upper right triangle are correlations for men. Costly service, non-costly service, and interpersonal helping were measured on a 4-point scale ranging from −1.5 = *really sure one of the boys* to +1.5 *= really sure one of the girls.* Communal stereotypes were measured on a 5-point scale ranging from −2 = *boys care a lot more* to +2 = *girls care a lot more*. ^*^ *p* < .05, ^**^ *p* < .01, ^***^ *p* < .001

**Table S8**

*Linear Mixed-Effects Regression on Adults’ Expectations about Helping*

|  | **Adults’ Expectations**  **about Helping** | | |
| --- | --- | --- | --- |
| *Predictors* | *χ*^2^ | *df* | *p* |
| Vignette Type | 2.06 | 2 | .357 |
| Woman | 31.65 | 1 | **< .001** |
| Vignette Type × Woman | 9.67 | 2 | **.008** |
| *N*_Participants_ | 192 | | |
| *N*_Observations_ | 576 | | |

*Note*. Wald chi-square tests for the fixed effects of the linear mixed-effects model regressing adults’ expectations about helping on their gender (Woman; women = 1, men = 0), the type of helping (Vignette Type; interpersonal, non-costly service, costly service), and their interactions.

**Figure S2**

*Adults’ Expectations about Helping Across Three Types of Scenarios: Interpersonal Helping, Non-Costly Service, and Costly Service*

*Note*. Shapes are predicted means; error bars are 95% confidence intervals. ** *p* < .01

**Table S9**

*Linear Mixed-Effects Regression on Adults’ Expectations about Helping, Adjusting for Their Communal Stereotypes*

|  | **Adults’ Expectations**  **about Helping** | | |
| --- | --- | --- | --- |
| *Predictors* | *χ*^2^ | *df* | *p* |
| Vignette Type | 2.10 | 2 | .349 |
| Woman | 15.86 | 1 | **< .001** |
| Communal | 14.30 | 1 | **< .001** |
| Vignette Type × Woman | 9.88 | 2 | **.007** |
| Vignette Type × Communal | 2.98 | 2 | .225 |
| Woman × Communal | 0.51 | 1 | .473 |
| Vignette Type × Woman × Communal | 0.08 | 2 | .962 |
| *N*_Participants_ | 192 | | |
| *N*_Observations_ | 576 | | |

*Note*. Wald chi-square tests for the fixed effects of the linear mixed-effects model regressing adults’ expectations about helping on their gender (Woman; women = 1, men = 0), the type of helping (Vignette Type; interpersonal, non-costly service, costly service), their communal stereotypes (Communal; higher values correspond to stronger feminine stereotypes about communality), and all interactions.

# Section 6: Communal Stereotypes

While not the focus of the present manuscript, we did measure children’s and adults’ communal stereotypes. Here, we report the findings across these samples and replicate Block et al. (2025).

**Table S10**

*Linear Regression on Participants’ Communal Stereotypes*

|  | **Children** | | | **Adults** | | |
| --- | --- | --- | --- | --- | --- | --- |
| *Predictors* | *b* | *SE* | *p* | *b* | *SE* | *p* |
| (Intercept) | 0.30 | 0.04 | **< .001** | 0.65 | 0.04 | **< .001** |
| Female | 0.45 | 0.09 | **< .001** | 0.37 | 0.07 | **< .001** |
| Age | −0.03 | 0.04 | .367 |  |  |  |
| Female × Age | −0.21 | 0.08 | **.007** |  |  |  |
| Observations | 192 | | | 192 | | |
| R^2^ / R^2^ adjusted | .157 / .143 | | | .125 / .120 | | |

*Note*. Estimates (with standard errors) and goodness-of-fit statistics for linear models regressing participants’ communal stereotypes (−2 = *boys care a lot more*, +2 = *girls care a lot more*) on the participants’ gender (Female; girls and women = 1, boys and men = 0). For child participants, we additionally include their age (Age; continuous) and interaction between the two. Predictors are mean-centered to facilitate interpretation of lower-order effects, including the intercept.

**Figure S3**

*Children’s (A) and Adults’ (B) Communal Stereotypes*

*Note*. Lines (A) and solid shapes (B) are model-predicted means, ribbons (A) and black lines (B) are 95% confidence intervals. Small shapes are individual datapoints (none of which are obscured by the figure legend in Panel A).

# Section 7: Costly Interpersonal Helping

We designed measures of costly helping for children and adults to complete at the end of the study (see “Real Costly Helping Behavior” in Section 1 above for exact wording). In retrospect, neither the child nor the adult measure of costly helping assesses *service*: In neither case are participants taking on work as a part of a group to the benefit of that group. Instead, they are asked whether they would provide help to the experimenter (an individual) at a cost to their time—an instance of costly interpersonal helping.

Further, the operationalization of costly helping differed across age groups in ways that complicate comparison. Children were asked if they would spend time counting up other children’s answers for no additional benefit; adults were asked if they would complete another study for no additional benefit. These tasks likely differ in their cost-benefit structure: Having access to other children’s answers is probably more enticing for children than completing an additional study is for adults, introducing an intrinsic benefit for children that offsets the time cost. This asymmetry makes it difficult to compare helping rates across age groups or to treat the measure as capturing the same construct in both samples.

We present the results here for openness, but we do not interpret either measure as reflecting participants’ likelihood to do service.

We preregistered three sets of models. First, a linear regression with helping regressed on participant gender, participant age, and their interaction. (We removed age for analyses of adults’ helping.) Second, a linear regression with participants’ own-gender costly service stereotypes interacting with all other terms in the model and a covariate for own-gender communal stereotypes. Third, a linear regression with the terms for own-gender communal stereotypes and own-gender costly service stereotypes swapped.

For children, there were no significant effects: Girls and boys were similarly likely to help, *p*s > .89, and this did not vary by their own-gender costly service stereotypes, *p*s > .06, or their own-gender communal stereotypes, *p*s > .27.

For adults, there was a significant main effect of participant gender: Women (observed range: 0–2; *M* = 0.42) were more likely to provide costly help to the experimenter than were men (*M* = 0.22), *β* = 0.32, *SE* = 0.14, *p* = .0301. This did not vary by their own-gender costly service stereotypes, *p*s > .14, or their own-gender communal stereotypes, *p*s > .33. However, when including these terms in the regression, the coefficient on participant gender was no longer significant. This attenuation could suggest that some of the gender differences in helping behavior reflect women’s associating service and/or communality with their own gender, though this interpretation is tentative: The stereotype terms themselves were not significant predictors of helping behavior, and with only 26% (49/191) of participants helping to any degree, the analysis is underpowered to detect these effects reliably.

**Table S11**

*Linear Regression on Participants’ Costly Interpersonal Helping*

|  | **Children** | | | **Adults** | | |
| --- | --- | --- | --- | --- | --- | --- |
| *Predictors* | *β* | *SE* | *p* | *β* | *SE* | *p* |
| (Intercept) | −0.00 | 0.07 | .961 | 0.00 | 0.07 | .992 |
| Female | −0.02 | 0.15 | .898 | 0.32 | 0.14 | **.030** |
| Age | 0.13 | 0.08 | .087 |  |  |  |
| Female × Age | −0.02 | 0.15 | .892 |  |  |  |
| Observations | 181 | | | 191 | | |
| R^2^ / R^2^ adjusted | .017 / −.000 | | | .025 / .019 | | |

*Note*. Estimates (with standard errors) and goodness-of-fit statistics for linear models regressing participants’ costly helping (standardized) on their gender (Female; girls and women = 1, boys and men = 0). For child participants, we additionally include their age (Age; continuous) and interaction between the two. Continuous variables are standardized (Help, Age) and dichotomous variables (Female) are mean-centered to facilitate interpretation of lower-order effects.

**Table S12**

*Linear Regression on Participants’ Costly Interpersonal Helping, as Predicted by Own-Gender Costly Service Stereotypes*

|  | **Children** | | | | **Adults** | | |
| --- | --- | --- | --- | --- | --- | --- | --- |
| *Predictors* | *β* | *SE* | *p* | *β* | | *SE* | *p* |
| (Intercept) | 0.02 | 0.08 | .745 | −0.05 | | 0.08 | .502 |
| Female | 0.05 | 0.17 | .785 | 0.40 | | 0.24 | .090 |
| Age | 0.16 | 0.08 | .050 |  | |  |  |
| Own-Costly | −0.04 | 0.08 | .623 | 0.12 | | 0.08 | .144 |
| Own-Communal | −0.08 | 0.09 | .385 | −0.11 | | 0.12 | .352 |
| Female × Age | −0.11 | 0.16 | .483 |  | |  |  |
| Female × Own-Costly | −0.31 | 0.17 | .061 | 0.24 | | 0.17 | .153 |
| Age × Own-Costly | 0.13 | 0.08 | .113 |  | |  |  |
| Female × Age × Own-Costly | −0.19 | 0.17 | .260 |  | |  |  |
| Observations | 181 | | | 191 | | | |
| R^2^ / R^2^ adjusted | .057 / .013 | | | .051 / .031 | | | |

*Note*. Estimates (with standard errors) and goodness-of-fit statistics for linear models regressing participants’ costly helping (standardized) on their gender (Female; girls and women = 1, boys and men = 0), own-gender costly service stereotypes (Own-Costly; higher values represent higher expectation for own gender engagement in service), and all interactions, with a covariate for own-gender communal stereotypes (Own-Communal; higher values represent higher expectation for own gender valuing communality). For child participants, we additionally include their age (Age; continuous). Continuous variables are standardized (Help, Age, Own-Costly, Own-Communal) and dichotomous variables (Female) are mean-centered to facilitate interpretation of lower-order effects.

**Table S13**

*Linear Regression on Participants’ Costly Interpersonal Helping, as Predicted by Own-Gender Communal Stereotypes*

|  | **Children** | | | **Adults** | | |
| --- | --- | --- | --- | --- | --- | --- |
| *Predictors* | *β* | *SE* | *p* | *β* | *SE* | *p* |
| (Intercept) | 0.01 | 0.09 | .880 | 0.07 | 0.12 | .557 |
| Female | 0.11 | 0.18 | .523 | 0.34 | 0.24 | .165 |
| Age | 0.07 | 0.09 | .461 |  |  |  |
| Own-Communal | −0.08 | 0.10 | .455 | −0.12 | 0.12 | .338 |
| Own-Costly | −0.09 | 0.08 | .286 | 0.14 | 0.08 | .106 |
| Female × Age | −0.02 | 0.18 | .932 |  |  |  |
| Female × Own-Communal | −0.08 | 0.20 | .702 | −0.18 | 0.24 | .463 |
| Age × Own-Communal | −0.01 | 0.10 | .938 |  |  |  |
| Female × Age × Own-Communal | 0.21 | 0.19 | .276 |  |  |  |
| Observations | 181 | | | 191 | | |
| R^2^ / R^2^ adjusted | .043 / −.001 | | | .044 / .023 | | |

*Note*. Estimates (with standard errors) and goodness-of-fit statistics for linear models regressing participants’ costly helping (standardized) on their gender (Female; girls and women = 1, boys and men = 0), own-gender communal stereotypes (Own-Communal; higher values represent higher expectation for own gender valuing communality), and all interactions, with a covariate for own-gender costly service stereotypes (Own-Costly; higher values represent higher expectation for own gender engagement in service). For child participants, we additionally include their age (Age; continuous). Continuous variables are standardized (Help, Age, Own-Costly, Own-Communal) and dichotomous variables (Female) are mean-centered to facilitate interpretation of lower-order effects.

# Section 8: Exploratory Robustness Checks

We additionally perform three exploratory analyses to check the robustness of the critical interaction between vignette type and participant gender in the main model (Section 3 Table S2). Specifically, whether this interaction holds and/or is moderated by the following factors: comprehension checks, order effects, and vignette.

First, we test for moderation by responses to comprehension check questions (see Table S14). Because we excluded any participants who failed two or more comprehension check questions and exclude the data from vignettes for any failed comprehension check, this analysis tests whether participants who responded incorrectly to one comprehension check (*n*_children_ = 55; *n*_adults_ = 16) pattern differently than those who responded correctly to all comprehension checks (*n*_children_ = 137; *n*_adults_ = 176). The pattern of results for fixed effects held (vignette type × participant gender interaction: *χ*²(2) = 16.39, *p* < .001). We find that this comprehension check term does not significantly predict or interact with any other terms to predict participants’ responses (*p*s > .07), suggesting that participants who failed one comprehension check are not driving or masking the effects reported in the main text.

Second, we test for order effects (see Table S15). In the present study, we randomized whether participants saw vignettes about costly or non-costly service first (with interpersonal vignettes always being shown last). Therefore, we included a term for whether costly service vignettes were shown first (costly first = 1, costly second = 0) and interacted it with all other fixed effects in the model. The key vignette type × participant gender interaction held (vignette type × participant gender interaction: *χ*²(2) = 16.19, *p* < .001) and was not moderated by order (*p*s > .19). There were, however, some other significant terms involving order: a two-way vignette type × order interaction (*χ*²(2) = 7.93, *p* = .019) and a three-way vignette type × participant age group × order interaction (*χ*²(2) = 6.22, *p* = .045^[1]^). These interactions suggested that, in general, participants were more likely to choose ‘one of the girls’ for the first vignettes they saw, with this effect being present for adults but not children (see Figure S4 for visualization).

Third and lastly, we examined whether the present effects hold when analyzing the data at the vignette level instead vignette-type level (see Table S16). We did this to examine whether any one vignette was driving the effects found in the manuscript. The pattern of results for fixed effects held (vignette type × participant gender interaction: *χ*²(2) = 12.06, *p* = .002). Further, we probed the ICC of the random intercepts of vignette and participant to examine whether either feature contributed significantly to variation in responses not explained by the fixed effects. Variance attributable to the vignette was negligible (ICC = .003), suggesting that responses varied very little across vignettes after accounting for the fixed effects in the model. For comparison, the variance attributable to participant-level clustering was much higher yet still small (ICC = .025), indicating that only about 2.5% of the variance in responses reflected differences between participants. Therefore, we are confident that the present findings are not attributable to any specific vignette behaving differently than the others.

**Table S14**

*Linear Mixed-Effects Regression on Participants’ Expectations about Helping, Adjusting for Whether They Passed All Comprehension Checks*

|  | **Expectations**  **about Helping** | | |
| --- | --- | --- | --- |
| *Predictors* | *χ*^2^ | *df* | *p* |
| Vignette Type | 0.51 | 2 | .776 |
| Female | 66.48 | 1 | **< .001** |
| Children | 2.45 | 1 | .118 |
| Checks | 3.78 | 1 | .052 |
| Vignette Type × Female | 16.39 | 2 | **< .001** |
| Vignette Type × Children | 1.02 | 2 | .600 |
| Female × Children | 4.29 | 1 | **.038** |
| Vignette Type × Checks | 0.15 | 2 | .930 |
| Female × Checks | 0.01 | 1 | .937 |
| Children × Checks | 0.07 | 1 | .788 |
| Vignette Type × Female × Children | 0.20 | 2 | .906 |
| Vignette Type × Female × Checks | 5.08 | 2 | .079 |
| Vignette Type × Children × Checks | 3.39 | 2 | .184 |
| Female × Children × Checks | 0.28 | 1 | .594 |
| Vignette Type × Female × Children × Checks | 0.30 | 2 | .859 |
| *N*_Participants_ | 384 | | |
| *N*_Observations_ | 1152 | | |

*Note*. Wald chi-square tests for the fixed effects of the linear mixed-effects model regressing children’s and adults’ expectations about helping on participants’ gender (Female; girls and women = 1, boys and men = 0), their age group (Children; children = 1, adults = 0), the type of helping (Vignette Type; interpersonal, non-costly service, costly service), whether they passed all four comprehension checks or only three (Checks; passed 4/4 checks = 1, passed 3/4 checks = 0), and all interactions.

**Table S15**

*Linear Mixed-Effects Regression on Participants’ Expectations about Helping, Adjusting for Order of Costly vs. Non-Costly Service Vignettes*

|  | **Expectations**  **about Helping** | | |
| --- | --- | --- | --- |
| *Predictors* | *χ*^2^ | *df* | *p* |
| Vignette Type | 0.51 | 2 | .773 |
| Female | 67.93 | 1 | **< .001** |
| Children | 1.03 | 1 | .310 |
| Costly First | 0.00 | 1 | .973 |
| Vignette Type × Female | 16.19 | 2 | **< .001** |
| Vignette Type × Children | 1.74 | 2 | .419 |
| Female × Children | 5.33 | 1 | **.021** |
| Vignette Type × Costly First | 7.93 | 2 | **.019** |
| Female × Costly First | 0.00 | 1 | .945 |
| Children × Costly First | 1.51 | 1 | .219 |
| Vignette Type × Female × Children | 0.08 | 2 | .959 |
| Vignette Type × Female × Costly First | 1.46 | 2 | .482 |
| Vignette Type × Children × Costly First | 6.22 | 2 | **.045** |
| Female × Children × Costly First | 1.24 | 1 | .265 |
| Vignette Type × Female × Children × Costly First | 3.26 | 2 | .196 |
| *N*_Participants_ | 384 | | |
| *N*_Observations_ | 1152 | | |

*Note*. Wald chi-square tests for the fixed effects of the linear mixed-effects model regressing children’s and adults’ expectations about helping on participants’ gender (Female; girls and women = 1, boys and men = 0), their age group (Children; children = 1, adults = 0), the type of helping (Vignette Type; interpersonal, non-costly service, costly service), whether costly or non-costly service vignettes were presented first (Costly First; costly vignettes presented first = 1, costly vignettes presented second = 0), and all interactions.

**Figure S4**

*Participants’ Expectations about Helping by Vignette Type, Participant Age Group, and Order of Costly vs. Non-Costly Service Vignettes*

*Note*. Shapes are predicted means; error bars are 95% confidence intervals.

**Table S16**

*Linear Mixed-Effects Regression on Participants’ Expectations about Helping (at the Vignette Level Rather than Averaging Vignettes of the Same Type)*

|  | **Expectations**  **about Helping** | | |
| --- | --- | --- | --- |
| *Predictors* | *χ*^2^ | *df* | *p* |
| Vignette Type | 0.31 | 2 | .857 |
| Female | 78.47 | 1 | **< .001** |
| Children | 1.97 | 1 | .161 |
| Vignette Type × Female | 12.06 | 2 | **.002** |
| Vignette Type × Children | 1.69 | 2 | .430 |
| Female × Children | 6.94 | 1 | **.008** |
| Vignette Type × Female × Children | 0.25 | 2 | .882 |
| *N*_Participants_ | 384 | | |
| *N*_Vignettes_ | 6 | | |
| *N*_Observations_ | 2245 | | |

*Note*. Wald chi-square tests for the fixed effects of the linear mixed-effects model regressing children’s and adults’ expectations about helping on participants’ gender (Female; girls and women = 1, boys and men = 0), their age group (Children; children = 1, adults = 0), the type of helping (Vignette Type; interpersonal, non-costly service, costly service), and their interactions.

1. Throughout the manuscript, we use *p* = .0303 as our threshold for significance. We interpret this three-way interaction at *p* < .05 as it was an exploratory analysis that came up in the review process.
